# Therapeutic Targeting of Autophagy in Pancreatic Ductal Adenocarcinoma

**DOI:** 10.3389/fphar.2021.751568

**Published:** 2021-11-30

**Authors:** Alexander G. Raufi, Nicholas R. Liguori, Lindsey Carlsen, Cassandra Parker, Liz Hernandez Borrero, Shengliang Zhang, Xiaobing Tian, Anna Louie, Lanlan Zhou, Attila A. Seyhan, Wafik S. El-Deiry

**Affiliations:** ^1^ Laboratory of Translational Oncology and Experimental Cancer Therapeutics, Warren Alpert Medical School, Brown University, Providence, RI, United States; ^2^ Hematology/Oncology Division, Department of Medicine, Lifespan Health System and Brown University, Providence, RI, United States; ^3^ Joint Program in Cancer Biology, Lifespan Health System and Brown University, Providence, RI, United States; ^4^ Cancer Center at Brown University, Providence, RI, United States; ^5^ Temple University, Lewis Katz School of Medicine, Philadelphia, PA, United States; ^6^ Pathobiology Graduate Program, Warren Alpert Medical School, Brown University, Providence, RI, United States; ^7^ Department of Surgery, Warren Alpert Medical School, Brown University, Providence, RI, United States; ^8^ Department of Pathology and Laboratory Medicine, Warren Alpert Medical School, Brown University, Providence, RI, United States

**Keywords:** autophagy, pancreatic cancer, MEK inhibitors, ONC212, chloroquine, Atg5, LC3, beclin 1

## Abstract

Pancreatic ductal adenocarcinoma (PDAC) is an aggressive disease characterized by early metastasis, late detection, and poor prognosis. Progress towards effective therapy has been slow despite significant efforts. Novel treatment approaches are desperately needed and autophagy, an evolutionary conserved process through which proteins and organelles are recycled for use as alternative energy sources, may represent one such target. Although incompletely understood, there is growing evidence suggesting that autophagy may play a role in PDAC carcinogenesis, metastasis, and survival. Early clinical trials involving autophagy inhibiting agents, either alone or in combination with chemotherapy, have been disappointing. Recently, evidence has demonstrated synergy between the MAPK pathway and autophagy inhibitors in PDAC, suggesting a promising therapeutic intervention. In addition, novel agents, such as ONC212, have preclinical activity in pancreatic cancer, in part through autophagy inhibition. We discuss autophagy in PDAC tumorigenesis, metabolism, modulation of the immune response, and preclinical and clinical data with selected autophagy modulators as therapeutics.

## Pancreatic Cancer

Pancreatic ductal adenocarcinoma (PDAC) is an aggressive disease characterized by early metastasis, late detection, and little progress towards effective treatment or cure. The vast majority of patients present with incurable unresectable or metastatic disease. Even in the 15–20% of patients who are candidates for, and ultimately undergo resection, recurrence ultimately occurs in 80%. Presently, the mortality-to-incidence ratio for PDAC remains amongst the highest of all malignancies and by 2030 PDAC is projected to be the second leading cause of cancer-related death in the United States (Miller et al., 2016). For individuals diagnosed with unresectable or metastatic PDAC, combination chemotherapy with mFOLFIRINOX or gemcitabine/nab-paclitaxel remains the standard of care. These regimens provide modest benefit, improving quality of life and median overall survival by several months, however, the 5-years overall survival is only 10% (Siegel et al., 2021). In light of this, identifying novel therapeutic agents to treat PDAC has become a major focus of research.

Although several mutations (e.g., *KRAS, TP53, SMAD4, CDKN2A*) are commonly identified in PDAC, the disease is genetically complex and development of targeted therapy has been slow. Only two targeted therapies have been approved to date: erlotinib, an EGFR inhibitor which improves in overall survival by approximately 2 weeks, and olaparib, a PARP inhibitor, which improves progression free survival by several months in germline BRCA2-mutated metastatic PDAC that has not progressed after 4 months of platinum containing chemotherapy (Moore et al., 2007; Golan et al., 2019). Unfortunately, aside from rare cases of microsatellite instability, immune checkpoint blockade has also had little to no impact on outcomes for patients diagnosed with PDAC. The lack of effective therapies has served as an impetus to further improve our understanding of pancreatic tumor biology in order to identify alternative treatment strategies.

**FIGURE 1:**
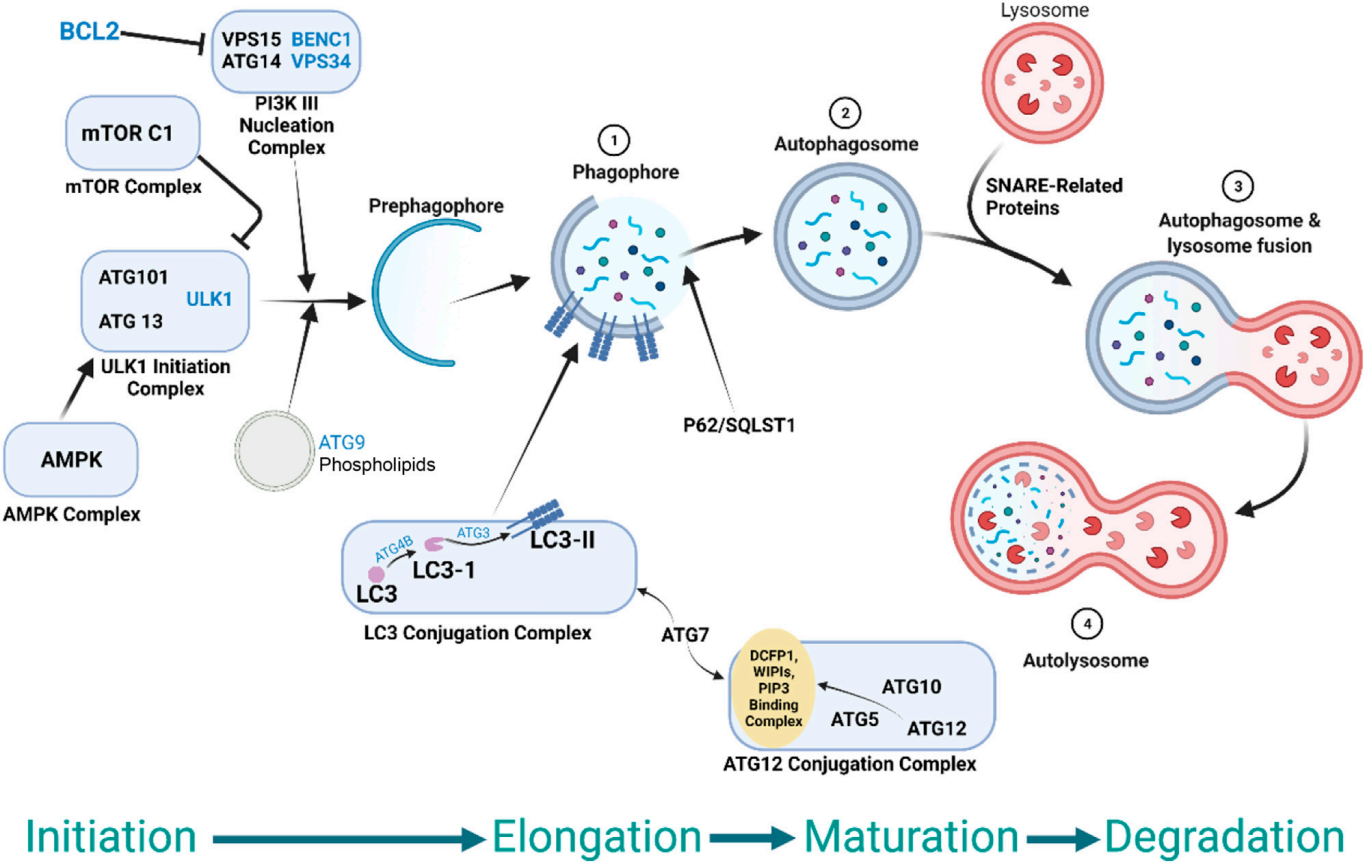
Schematic depiction of the autophagy pathway and its regulation by various signaling molecules, autophagosomes, and lysosomes in cell biology. Autophagosome formation is a complex process that involves several phases: Autophagosome initiation which involves ULK1 complex and the class III phosphatidylinositol 3-kinase (PI3K) complex and other protein complexes. The elongation step involves the action of two ubiquitin-like conjugation systems and requires the conjugation of LC3 to the phosphatidylethanolamine, a form called LC3-II, allowing the expansion of the initial membrane and confining a portion of the cytosol. The maturation and degradation step involve autophagosome closure, the fusion of the autophagosome with the lysosome to form the autolysosome, and degradation step mediated by lysosomal enzymes which degrade the proteins and other substrates in the autolysosome. More information on autophagosomes and autophagy process can be found in the literature (Reggiori and Ungermann, 2017).

Autophagy is a complex, evolutionarily conserved process through which proteins and organelles are recycled for use as alternative energy sources. Although typically upregulated during states of cellular stress or starvation, tumor cells can also take advantage of this process to maintain homeostasis. In this review we will focus on macro-autophagy, which refers to the removal of cytoplasmic components through autophagosome-delivery of organelles to lysosomes for degradation (Figure 1) (Mizushima et al., 2011). This process is required for cell survival, homeostasis, and can be upregulated through multiple cell signaling pathways. In cancer, it is thought to play a role in tumor cell survival and resistance to chemotherapy, and hence represents an area of therapeutic development.

We will discuss the role autophagy plays in PDAC tumorigenesis and metabolism, modulation of the immune response, as well as both preclinical and clinical data with select autophagy modulators.

## Autophagy is Upregulated in PDAC

Although the precise role of autophagy in PDAC is incompletely understood, increased basal levels of autophagy have been reported. Using GFP-LC3 puncta as an indicator of cells undergoing autophagy, Yang et al. demonstrated increased autophagic flux in eight PDAC cell lines (Yang et al., 2011). The authors further supported these findings by measuring levels of microtubule-associated protein 1 light chain 3 (LC3), more specifically the conversion of LC3-I to LC3-II. Previous work established an interaction between LC3 and autophagosome membranes, notably in PDAC (Fujii et al., 2008). Yang et al. noted increased levels of LC3-II in PDAC cells lines, relative to control normal pancreatic ductal cells, a finding that was not reproducible in select lung or breast cancer cell lines, suggesting that this may be a unique feature of PDAC. Given that autophagy is a dynamic process, elevations in LC3-II could suggest a block in later stages of autophagy, such as impaired autophagosome degradation, and not exclusively upregulation. Therefore, an analysis of long-term protein degradation using a GFP-Neo fusion protein was performed (Klionsky et al., 2008). Over a 2-day period, 8988T PDAC cells were examined and were noted to have a significant reduction in levels, further supporting increased autophagic flux. The authors were also able to restore GFP-Neo levels with the autophagy inhibitor chloroquine. Finally, they showed that chloroquine also reduces PDAC cell proliferation *in vitro*, suggesting a possible novel approach to therapy.

Several additional components of the autophagy pathway have been identified as key mediators in governing PDAC cell proliferation. ATG5, for example, is a ubiquitin-related protein shown to be necessary for autophagosome expansion and completion (Figure 1) (Levine and Kroemer, 2008). Selective siRNA-mediated knockdown of ATG5 notably reduced 8988T PDAC cell proliferation by greater than 50% (Yang et al., 2011). The MiTF family of transcription factors (MiTF, TFE3, and TFEB) have also been implicated as drivers of autophagy in PDAC cells (Rouschop et al., 2010). Upon nuclear import, these transcription factors drive increased expression of catabolic lysosomal genes and gene set enrichment analyses indicate a strong relationship between expression of MiT/TFE factors and autophagy in PDAC (Rouschop et al., 2010). Furthermore, MITF, TFE3, or TFEB knockout leads to downregulation of CLEAR (Coordinated Lysosomal Expression and Regulation)-carrying genes in PDAC cells, leading to reduction in proliferation and growth of PDAC tumor cells (Rouschop et al., 2010).

In the setting of amino acid starvation, unc5-like autophagy activating kinase 1 (ULK1) is known to play an indispensable role in driving autophagy. It is primarily regulated by nutrient-sensing kinases such as mammalian target of rapamycin (mTOR) complex-1 (mTORC1) and AMPK (Kim et al., 2011; Shang et al., 2011; Wong et al., 2015). When starvation-levels of amino acids are detected, mTORC1 is suppressed and ULK1 is phosphorylated inducing autophagy (Kim et al., 2011; Shang et al., 2011; Wong et al., 2015). Interestingly, starvation appears to be a more profound driver of autophagy than direct inhibition of mTORC1, suggesting that alternative pathways also play a role (Wong et al., 2015). Furthermore, cells with high levels of autophagy also have increased phosphatase activity, including phosphatase PPA2. This enzyme dephosphorylates ULK1 at S637 reducing levels of autophagy (Wong et al., 2015).

Given that the vast majority of PDAC cases have constitutive activation of KRAS, the effects of the MAPK pathway on autophagic flux is of particular interest. Although poorly defined, it is unlikely that constitutive MAPK signaling is solely responsible for driving increased basal levels of autophagy in PDAC. In fact, several studies have reported that inhibition of the MAPK cascade leads to increased autophagy which will be described in further detail later (Bryant et al., 2019; Kinsey et al., 2019). As described above, activation of the MAPK pathway is expected to promote phosphorylation and cytoplasmic retention of the transcription factors TFEB and TFE3, and hence a reduce expression of autophagy promoting genes.

In summary, high rates of basal autophagy in PDAC is regulated by multiple mechanisms and each of these processes represents a unique target for further investigation.

## Autophagy and PDAC Carcinogenesis

The precise role autophagy plays in PDAC tumorigenesis is complicated by several conflicting studies that have shown that autophagy can lead to both promotion and inhibition of tumor development. A tumor-promoting mechanism of autophagy has been described in mice with heterozygous deletions of mammalian Beclin1. Deletion of this key autophagy promoting enzyme results in the development of malignant neoplasms in various organs in mice (Qu et al., 2003; Yue et al., 2003). Another partial autophagy phenotype, ATG5^+/−^, leads to increased tumor formation and metastasis but this is not observed in mice completely deficient of autophagy (ATG^−/−^) which spontaneously developed only benign liver tumors and increased acinar-to-ductal metaplasia (Takamura et al., 2011; Görgülü et al., 2019). It has been suggested that autophagy is a relatively weak tumor suppressor yet at the same time it is necessary for the progression of benign tumors to malignancy (Takamura et al., 2011). There is also evidence suggesting that defects in autophagy lead to increased dysfunctional or damaged mitochondria in tumor cells and impaired tumorigenesis (White, 2015). This implies that autophagy may induce tumorigenesis and disease by preserving the integrity and quality of mitochondria and also by supplementing essential substrates for mitochondrial metabolism (White, 2015). Autophagy may also promote tumorigenesis by suppressing induction of the p53 tumor suppressor protein and maintaining metabolic function of mitochondria, enabling cancer cells to survive environmental stresses (White, 2015). Further study is required to bring clarity to our understanding of autphagic recycling of substrates, the identity of specific substrates, and the metabolic pathways and functions that they are used for.

Using a KRAS-driven lung cancer model, Guo et al. found that homozygous deletion of ATG7 reduced tumor burden and proliferation of tumor cells (Guo et al., 2016). ATG5, another member of the ATG family, was shown to increase PanIN but not PDAC formation in a genetically engineered PDAC mouse model with mutant KRAS and a single Trp53 allele. Chloroquine or hydroxychloroquine treated PDAC cell lines and patient derived xenograft models led to decreased proliferation, increased DNA damage and apoptosis (Yang et al., 2014). Interestingly, ATG7 deletion in a similar KRAS mutant/Trp53null model of lung cancer showed reduced tumor burden (Karsli-Uzunbas et al., 2014). These studies support the role that autophagy plays in carcinogenesis and in maintaining tumor growth and proliferation.

## Autophagy and Metabolomics

As discussed in a recent review, cellular metabolism and autophagy are two interconnected cellular processes (Piffoux et al., 2021). A hallmark of tumor metabolism is the preferred use of aerobic glycolysis over oxidation of glycolytic pyruvate to produce both energy and lactate, the latter of which serves as a substrate for nucleic acid, protein, and lipid production. While aerobic glycolysis is inefficient in terms of energetics, it serves as a mechanism to promote growth, survival, and proliferation in tumor cells. This phenomenon of increased glucose uptake and fermentation of glucose to lactate is observed even in the presence of completely functioning mitochondria and is known as the Warburg Effect (Vander Heiden et al., 2009; Liberti and Locasale, 2016). Because autophagy degrades proteins and organelles to create new substrates it is integrally connected with tumor metabolism (Vander Heiden et al., 2009). It has been reported that oncogene ablation-resistant pancreatic cancer cells depend on mitochondrial function and that resistance to KRAS-targeted therapy might be mediated by a subset of tumor cells that depend on oxidative phosphorylation for survival instead of the classic Warburg effect (Viale et al., 2014). Oxidative phosphorylation is highly dependent on mitochondrial respiration, and genes involved in this process, as well as autophagy- and lysosome-related genes, were found to be upregulated in surviving cells. However, upregulation of autophagy in surviving cells is likely only one side of a transcriptional program which supplies tumor cells with nutrients (Perera et al., 2015). Collectively, autophagy has a role in maintaining sufficient supplies of energy and nutrient to tumors *via* tumor-cell-autonomous, stromal and systemic autophagy.

Autophagy induction is not only triggered by nutrient deficiency but also by low oxygen levels. Cellular adaptation to hypoxic conditions involves multiple mechanisms, such as upregulation of the unfolded protein response (UPR) (Rouschop et al., 2010). Hypoxia has been shown to increase transcription of the essential autophagy genes MAP1LC3B and ATG5 *via* the transcription factors ATF4 and CHOP, respectively. Notably, MAP1LC3B and ATG5 are not required for initiation of autophagy but are involved in phagophore expansion and autophagosome formation. Furthermore, autophagy and MAP1LC3B induction have been shown to mostly occur in hypoxic regions of tumor xenografts. Pharmacological inhibition of autophagy sensitizes human tumor cells to hypoxia and decreases the proportion of viable hypoxic tumor cells and sensitizes tumor xenografts to irradiation. Collectively, these data suggest that the UPR is an important mediator of the hypoxic tumor microenvironment and that it contributes to resistance to treatment through its ability to facilitate autophagy.

Hypoxia is involved in tumorigenesis, associated with altered metabolism, abnormal vascularization, resistance to chemo/radiotherapy, and increased cancer cell stemness and may even promote metastasis (Wilson and Hay, 2011; Yun and Lin, 2014; Horsman and Overgaard, 2016; Minassian et al., 2019). In response to hypoxia, the transcription factor hypoxia-inducible factor 1α (HIF1α), activates a variety of target genes that are involved in altered metabolism, cell survival and tumor progression (Kaelin, 2011; Masson and Ratcliffe, 2014; Chen and Sang, 2016). Both hypoxia and anoxia, with oxygen concentrations <3% and <0.1%, respectively, cause autophagy through a variety of different mechanisms (Kroemer et al., 2010). Hypoxia-induced autophagy depends on hypoxia-inducible factor, HIF, while anoxia-induced autophagy is HIF-independent (Majmundar et al., 2010; Mazure and Pouysségur, 2010). HIF is a heterodimer of a constitutive ß subunit and an oxygen-regulated α subunit that only becomes stabilized (and hence expressed) when oxygen concentration declines below a threshold of ∼5%. Under moderate hypoxia (1–3% oxygen), HIF activates the transcription of *BNIP3* and *BNIP3L* (NIX), two BH3-only proteins that can disrupt the inhibitory interaction between Beclin 1 and Bcl-2 (Bellot et al., 2009). Moreover, BNIP3L, which often is present at the outer surface of mitochondria, possesses a WXXL motif that binds to LC3 and its homolog GABARAP (Novak et al., 2010), thereby targeting mitochondria for autophagic destruction. The transcription of *BNIP3* is also upregulated by the transcription factor FOXO3, on condition that it is deacetylated by Sirt1 (Kume et al., 2010).

Under severe hypoxia or anoxia, additional pathways including the protein DJ-1, the autocrine stimulation of a PDGFR-dependent pathway, the stimulation of AMPK through metabolic stress, and the UPR of the ER have been demonstrated to play role in hypoxia-induced autophagy (Mazure and Pouysségur, 2010). Hypoxia-mediated upregulation of autophagy also requires phosphorylation of eIF2α mediated by PERK (see below), further highlighting the significance of the phosphorylation of eIF2α as a universal autophagy regulator (Rouschop et al., 2010). Lastly, hypoxia has been shown to upregulate the transcription of the key autophagy genes, *LC3* and *Atg5*, *via* ATF4 and CHOP transcription factors, respectively, which are both regulated by PERK (Rouschop et al., 2010).

## Autophagy and the Integrated Stress Response

The integrated stress response (ISR) is an evolutionarily conserved cellular stress response in eukaryotic organisms that inhibits global protein biosynthesis and activates the expression of specific genes in response to extrinsic environmental factors and intrinsic pathophysiological stresses (Pakos-Zebrucka et al., 2016). Extrinsic stress factors include hypoxia, starvation (e.g., amino acid deprivation, glucose deprivation), viral infection, and presence of oxidants. One of the primary intrinsic factors is endoplasmic reticulum (ER) stress which results from increased levels of unfolded proteins and polypeptides in the ER. It is now well established that oncogene activation can also activate the ISR. Activation of the ISR will either stimulate the expression of specific genes to restore cellular homeostasis by resolving cellular damage caused by these stressors, or, if unable to restore homeostasis, activate programmed cell death (apoptosis) (Pakos-Zebrucka et al., 2016).

Many of the stress signaling pathways converge on eIF2α. Phosphorylation of this transcription factor subsequently initiates the ISR, but outcome of ISR activation can be quite different and depends not only by the type of the stressor but also its extent and severity. This influences the duration of the phosphorylation of eIF2α as well as translation of *ATF4* and other bZIP transcription factors (Dey et al., 2010; Guan et al., 2014). For example, a short duration of ISR activity appears to be an adaptive, pro‐survival response to various stresses aimed at overcoming the stress and restoring homeostasis, whereas activation of ISR for an extended period can induce the cell to programmed cell death (Rutkowski et al., 2006). However, this dual action of eIF2α phosphorylation requires further elucidation.

It has been widely accepted that the ISR can regulate cell survival and cell death pathway *via* induction of autophagy which facilitates the degradation of unfolded proteins, polypeptides or protein aggregates, and damaged organelles. As a result, autophagy restores depleted amino acids pool for protein synthesis and reenergizes a starved cell restoring homeostasis. Although mechanisms by which phosphorylated eIF2α induces autophagy are still being explored, similar extrinsic and intrinsic stress signals leading to phosphorylation of eIF2α have been shown to activate autophagy. For example, ER stress-induced phosphorylation of eIF2α phosphorylation has been shown to upregulate a number of autophagy receptors such as *SQSTM1, NBR1*, and *BNIP3L via* PERK (Deegan et al., 2013). Furthermore, pharmacologic suppression of PERK represses transcriptional upregulation of these autophagy receptors (Deegan et al., 2015). Likewise, eIF2α phosphorylation-mediated by PERK upregulates the conversion of ATG12 and LC3 as a result of expression of polyQ72 aggregates, which is an important phase for the formation of autophagy (Kouroku et al., 2007). Consequently, the PERK-driven Unfolded Protein Response (UPR) regulates autophagy process from induction, to vesicle nucleation, phagophore elongation, and maturation (Deegan et al., 2013). The UPR, which is initiated in the setting of accumulation of misfolded proteins in the ER, is predominantly an adaptive response to the activation of the ISR. UPR protects cancer cells during hypoxia through regulation of the autophagy genes MAP1LC3B and ATG5 (Rouschop et al., 2010) and this is facilitated by PERK phosphorylation of eIF2α. On the other hand, elimination of PERK signaling or expression of mutant eIF2α S51A which cannot be phosphorylated under hypoxia decreases the transcription of *MAP1LC3B* and *ATG5* (Rouschop et al., 2010).

Amino acid deprivation in cancer cells also promotes the phosphorylation of eIF2α *via* GCN2, a protein essential for the activation of autophagy (Ye et al., 2010). GCN2 knockout cells exhibit decreased LC3 expression, whereas cells with mutant the eIF2α S51A cannot induce LC3 processing (Ye et al., 2010). Similarly, phosphorylation of eIF2α at S51 was found to be essential for regulation of autophagy induced by amino acid starvation in yeast and mouse embryonic fibroblasts (MEFs) (Tallóczy et al., 2002).

Critically, ATF4, which is essential for activation of autophagy, is downstream of eIF2α (Kroemer et al., 2010). ATF4 activation in response to stress signals induced by amino acid deprivation upregulates several autophagy genes transcriptionally including *Atg3, Atg5, Atg7, Atg10, Atg12, Atg16, Becn1, Gabarap, Gabarapl2, Map1lc3b,* and *Sqstm1* (B'Chir et al., 2013). In addition, ATF4 medicates REDD1, which represses the activity of mTORC1 under conditions of ER stress or amino acid deprivation, subsequently inducing autophagy (Whitney et al., 2009; Rzymski et al., 2010; B'Chir et al., 2013; Dennis et al., 2013; Deegan et al., 2015). Notably, several autophagy genes may have a varying magnitude of dependence on ATF4 and CHOP signaling and the transcriptional activation of these genes is controlled by the ratio of ATF4 and CHOP proteins that are bound to a particular promoter suggesting that the level of expression of autophagy genes depend on the needs of the cell (B'Chir et al., 2013).

Notably, a conditionally active form of the eIF2α kinase PKR functions upstream of PI3K and activates the Akt/PKB-FRAP/mTOR pathway leading to the phosphorylation of ribosomal protein S6 kinase 1 (S6K1) and eukaryotic initiation factor 4E binding protein 1 (4E-BP1) and that stimulation of PI3K signaling antagonizes the apoptotic and protein synthesis suppressive effects of the conditionally active PKR (Kazemi et al., 2007; Showkat et al., 2014). Furthermore, pharmacologic suppression of proteasome function with antineoplastic agent bortezomib results in depletion of amino acids in the ER required for protein synthesis leading to the activation of the ISR *via* GCN2 stress sensor (Suraweera et al., 2012). These findings suggest that proteasome inhibition has a role on survival signaling by the ISR. Moreover, amino acid depletion mediated by proteasome inhibition also induces autophagy through mTOR in an attempt to restore amino acid homeostasis (Suraweera et al., 2012), whereas, supplementation of essential amino acids depleted by the inhibition of proteasome function inhibition impairs the phosphorylation of eIF2α and down-regulates autophagy (Suraweera et al., 2012). Thus, depletion of amino acids by proteosome inhibition forms a connection between ISR activation and activation of autophagy to sustain cell survival.

Therefore, PERK, which facilitates the phosphorylation of eIF2α and inducing the ISR, acts alongside the different components of the UPR, IRE1, and ATF6 to suppress proteotoxicity induced by misfolded proteins and polypeptides. This is accomplished by upregulating the transcription of genes that stimulate proper protein folding and increase degradation of misfolded or aggregated proteins (Harding et al., 2000; Liu et al., 2000), as such, the cross talk between the various components of the UPR regulates the cellular outcome (Szegezdi et al., 2006). The ISR-mediated cell survival during ER stress indicates that ATF4 acts as a hub connecting PERK‐mediated translational control with IRE1‐ and ATF6‐mediated gene expression (Ron, 2002). Strikingly, the relative extent of PERK and IRE1 signaling appears to be critical for determining the cell fate, with the constant stimulation of PERK leading to activation of programmed cell death (i.e., apoptosis) and extended duration of activation of IRE1 leading to cell survival (Lin et al., 2007; Lin et al., 2009).

## Autophagy as a Mechanism of Resistance to Anticancer Therapy

Tumor cell activation of autophagy has been described as a potential mechanism of resistance to anticancer therapy. This is supported by several *in vitro* studies demonstrating that further augmentation of autophagic flux results in increased resistance to chemotherapy, resistance that can be overcome with inhibition of autophagy (Sotelo et al., 2006; Carew et al., 2007; Firat et al., 2012; Hu et al., 2012; Zou et al., 2012). In pancreatic cancer, inducing autophagy through upregulation of receptor for advanced glycation end products (RAGE) increases resistance to chemotherapy *in vivo* (Kang et al., 2010). Although further studies are necessary to elucidate precise mechanisms of resistance, autophagy-induced activation of several common cell signaling pathways have been described. These include epidermal growth factor receptor (EGFR), PI3K/AKT/mTOR, MAPK, and p53 pathways. Han et al. demonstrated that inhibiting EGFR with either gefitinib or erlotinib not only activates autophagy but also serves as a cytoprotective mechanism in human lung cancer. They further combined these tyrosine kinase inhibitors with various autophagy inhibitors or siRNAs targeting ATG5/7 and demonstrated enhanced cell killing (Han et al., 2011). As described earlier in this review, inhibition of the MAPK pathway also leads to up-regulation of autophagy and has been proposed as a mechanism of drug resistance. Furthermore, PI3K/mTOR inhibitors have been shown to induce protective autophagy in malignant peripheral nerve sheath tumor (MPNST) cells; however, pretreatment with chloroquine or bafilomycin consistently reverses this, potentially representing a treatment strategy in this difficult to treat sarcoma subtype (Ghadimi et al., 2012). The reciprocal interaction between autophagy and p53 may also have important implications for cancer therapy. Autophagic flux increases suppression of p53 while p53 activates autophagy (White, 2016). Autophagy inhibition alone is unlikely sufficient to overcome autophagy-induced resistance to anticancer therapy, however, a deeper understanding of autophagy in this setting may lead to new therapeutic approaches.

## ONC212, Autophagy and PDAC

Our work unraveling cell death pathways (Carneiro and El-Deiry, 2020) as an approach to understand and therapeutically target human cancer led us to discover TRAIL receptor DR5 as a p53 target gene (Wu et al., 1997). We discovered that the Tumor Necrosis Factor-Related Apoptosis-Inducing Ligand (TRAIL), the ligand for DR5 in the extrinsic cell death pathway is also a p53-regulated gene (Kuribayashi et al., 2008). We performed screening for TRAIL-inducing compounds in 2007 and discovered TRAIL-Inducing Compound #10 (TIC10), later published in 2013 (Allen et al., 2013). TIC10 activated the TRAIL gene in a p53-independent manner that involved dual inhibition of ERK and Akt and nuclear translocation of Foxo3a to bind and transactivate the TRAIL gene (Allen et al., 2013). TIC10 was advanced to clinical trials as ONC201 (Stein et al., 2017). We discovered that ONC201/TIC10 activates the integrated stress response (ISR) through kinases HRI and PKR leading to eIF2-alpha phosphorylation, activation of ATF4, CHOP, and DR5 (Kline et al., 2016). We found that ONC201 targets cancer stem cells (Prabhu et al., 2015) and activates an immune response involving natural killer (NK) cells (Wagner et al., 2018). We collaborated with Provid and Oncoceutics to synthesize and test ONC201/TIC10 analogues and uncovered ONC212 as a potent analogue (Wagner et al., 2017).

ONC212 appeared to have efficacy against PDAC cells and xenografted tumors *in vivo* (Lev et al., 2017). ONC212 was found to target the integrated stress response and activate the TRAIL pathway. Moreover, the compound appears to act through a mechanism involving mitochondrial caseinolytic protease ClpP which targets degradation of multiple mitochondrial proteins including respiratory chain proteins involved in oxidative phosphorylation (Ferrarini, 2021). The mitochondrial stress signals the integrated tress response leading to cell death and also inhibits autophagy in pancreatic cancer (Ferrarini, 2021). As efforts are underway to bring ONC212 to clinical trials, we have been exploring combinations with ONC212 in pancreatic cancer (Jhaveri, 2020; Raufi, 2021). In particular, ONC212 appears to synergize with MEK inhibitors against PDAC cell lines, in part through effects involving autophagy inhibition (Raufi, 2021).

## Autophagy, Immune Cell Function and Response to Immune Checkpoint Blockade

PDAC is characterized by a unique and complex tumor immune microenvironment comprised of distinct stromal and tumor compartments. The stromal compartment contains cancer associated fibroblasts (CAFs), as well as both innate and adaptive immune cells. Autophagy is necessary for immune cell function, differentiation, and survival and therefore a thorough understanding of the impact of autophagy modulating agents on these cells is essential to developing new therapies.

Autophagy is required for pluripotent hematopoietic stem cell (HSC) survival and differentiation (Mortensen et al., 2011). HSCs give rise to monocytes, which differentiate into macrophages with phagocytic and cytokine production capabilities. Autophagy has been shown to be essential for monocyte survival as well as their differentiation into macrophages (Jacquel et al., 2012; Zhang et al., 2012). In mature macrophages, autophagy plays a role in LC3-mediated phagocytosis, a form of non-canonical autophagy that promotes immune tolerance (Cunha et al., 2018). The breakdown of biomolecules during autophagy also mediates antigen presentation by dendritic cells (Li et al., 2012; Germic et al., 2019). Interestingly, autophagy inhibition-mediated tumor regression can be hindered by macrophage depletion in an autochthonous mouse model of PDAC, suggesting an essential role of the innate immune system in tumor cell killing (Yang et al., 2018).

Autophagy is also essential for adaptive immune cell function, as it supports T cell renewal, differentiation, and homeostasis. In the thymus, negative selection of CD4^+^ T cells is at least partially directed by autophagy and the transition of CD4^−^CD8^−^cells to CD4^+^CD8^+^ cells is associated with maximum activation of autophagy, though its explicit role in this transition is incompletely understood. Autophagy also mediates T cell survival and differentiation outside the thymus. Upon autophagy inhibition, T cells accumulate organelles and shift their metabolism from oxidative phosphorylation to glycolysis. Cells that generate energy predominantly through oxidative phosphorylation [memory T cells, T regulatory cells (T-regs)] are particularly vulnerable to autophagy inhibition. The vulnerability of T-regs to autophagy inhibition is further enhanced due to their dependence on high levels of autophagy (Clarke and Simon, 2019). However, autophagy inhibition also degrades extracellular ATP and attracts T-regs. This mechanism likely plays an important role *in vivo*, as triggering autophagy in lung tumor-bearing mice improved the efficacy of chemotherapy and this was at least partially mediated by a reduction of tumor-infiltrating T-regs (Pietrocola et al., 2016).

Interest in immune checkpoint blockade (ICB) has increased in recent years following clinical success in treating various malignancies. Single agent ICB has had little to no impact on outcomes in patients with PDAC. This may be partly due to the immunosuppressive components of the tumor immune microenvironment therefore there is much interest in identifying combination treatments that improve responses to ICB (Bian and Almhanna, 2021). A recent compelling study reported that autophagy promotes immune evasion of PDAC *via* MHC-I degradation, and that autophagy inhibition and ICB synergize in mice to reduce tumor burden (Yamamoto et al., 2020). Similar observations in other cancer types support these findings. For example, mice with metastatic liver tumors experience an enhanced response to high dose IL-2 when combined with an autophagy inhibitor (Liang et al., 2012), and impairment of autophagy in mice with colon or breast tumors improved response to ICB therapy (Young et al., 2020). Together, these findings suggest a role of autophagy in limiting the response of immunotherapies such as ICB across cancer types and provide an exciting new direction for investigating combination treatments for PDAC and other cancers.

## Preclinical Studies in PDAC

The relationship between autophagy and tumor progression is complex. First, autophagy has been shown to suppress cancer initiation in many models. As described above, Rosenthal et al. showed that genetically modified mice with loss of autophagy genes Atg5 or Atg7 showed increased benign pancreatic cell tumor formation, but with lack of progression to malignant disease. Other genetically-modified mouse models have shown similar results in liver (Takamura et al., 2011) and lung (Strohecker et al., 2013) tumors. Additionally, there is evidence that once the growth of a malignant tumor has been initiated, autophagy promotes tumor progression. Degenhardt et al. explored the impact of autophagy on the tumor immune microenvironment and showed that autophagic activity is increased in the hypoxic tumor microenvironment, which ultimately leads to increased degradation of waste products resulting in decreased inflammation and increased tumor cell survival. This was further supported by Guo et al. who found that autophagy knock-out xenografts in a KRAS-activated mouse model showed reduced tumor growth and also exhibited an increased immune response, leading to the development of immune-driven pathologies, such as pneumonia (Guo et al., 2013). Levy et al. explored the potential role of this hyperactivated immune response in reduced tumor induction and growth in an autophagy knock-out model, and postulated that reduced induced autophagy in T cells may lead to more T cell-induced tumor cell killing (Mulcahy Levy and Thorburn, 2020). Lastly, evidence suggesting that autophagy is important for malignant cell growth can also be found at the genetic level. Transcriptome analysis has shown that core autophagy proteins highly conserved in cancer (Lebovitz et al., 2015) and that many of the transcription factors that promote autophagy are oncogenes (Roczniak-Ferguson et al., 2012).

Early pre-clinical investigations focused on the use of hydroxychloroquine and chloroquine, which act through inhibiting lysosomes, which in part leads to degradation of autophagosomes and endosomes (Dolgin, 2019). PDAC is an attractive solid tumor for autophagy inhibition, as autophagy is known to be increased in pancreatic cancer, and has been shown to correlate to poorer patient outcomes (Fujii et al., 2008). Friboes et al. showed that treatment of a malignant pancreatic cancer line with chloroquine lead to decreased cell viability and decreased levels of autophagy (Frieboes et al., 2014). Yang et al. showed decreased tumor progression in an *in vitro* model when cells grown from pancreatic cancer tumors grown in genetically modified mice were treated with chloroquine (Yang and Kimmelman, 2014). Because the mechanism of action of chloroquine and hydroxychloroquine is targeted at lysosomes, and therefore not specific to the inhibition of autophagy, it is difficult to determine to what degree autophagy inhibition actually contributes to their overall mechanism of action in cancer therapy. In 2014 Maes et al. examined the use of hydroxychloroquine and chloroquine against melanoma tumor cells in an *in vivo* model and showed that treatment with these drugs leads to a normalization of the organization of tumor vessel and function, thereby decreasing hypoxia and increasing delivery of other drugs, which could certainly contribute to their antitumor effect (Maes et al., 2014). Another small molecule target for autophagy inhibition is the molecule of the PI3K class III that is known to be important in the promotion of autophagy, and has been shown to be effective at blocking autophagy *in vivo* (Dowdle et al., 2014). Ronan et al. developed an inhibitor specific to this molecule that was shown to act synergistically with everolimus in lung and renal cancer *in vitro* (Ronan et al., 2014), and Honda et al. discovered an inhibitor shown to be effective against colorectal cancer as monotherapy in an *in vivo* model (Honda et al., 2016). This molecule also has issues with specificity. In addition to contributing to the activation of autophagosomes, it is also involved in endocytic and vesicular function, and therefore has produced concern for toxic off-target effects (Dolgin, 2019). Alternatively, many investigators are focusing in on the autophagy activating kinase ULK1. Lazarus et al. performed a structural activity relationship analysis of ULK1 in order to identify binding sites of the molecule most ideal structure of a drug to bind to and inhibit these sites (Lazarus et al., 2015; Lazarus and Shokat, 2015). Egan et al. went further to discover a specific substrate that exhibits potent and highly selective inhibition of ULK1 in an *in vitro* model, and showed that it induced increased cell death in glioblastoma and lung cancer cells when used in concert with mTOR inhibition (Tang et al., 2017).

As autophagy has been shown to be important in both blocking the initiation of tumor formation as well as potentiating the spread of tumors when growth has already been initiated, there has been interest in studying autophagy-activating drugs to treat PDAC. mTOR inhibitors have therefore been studied in several but have been shown to only lead to a cytostatic effect. In a review from 2019, Tian et al. (2019) postulate that this result is due to the ability of mTOR inhibitors to lead to optimization of the tumor microenvironment, and that this could be inhibited with the addition of an autophagy inhibitor, which could explain synergy seen in pre-clinical models that have examined dual therapy with mTOR inhibitors and autophagy inhibitors, such as the results that Ronan et al. saw when combining everolimus with and VSP34 inhibitor.

As previously mentioned, ONC212 is a novel potent imipridone analogue with preclinical activity against PDAC in multiple *in vivo* models, biochemical evidence of autophagy inhibition, and synergistic activity when combined with MEK inhibitors (Lev et al., 2017; Wagner et al., 2017; Ferrarini, 2021; Raufi, 2021). As p53 mutations are common in human cancer, including PDAC, we have pursued therapeutic targeting of tumors with mutant p53 (Wang et al., 2006; Bassett et al., 2008; Hernández Borrero and El-Deiry, 2021). We previously reported that a p53 pathway restoring small molecule, CB002, induces morphological changes of autophagy and modulates LC3B expression in a manner that requires pro-apoptotic Noxa induction (Richardson et al., 2017; Hernandez-Borrero et al., 2018). Our recent results suggest that in addition to partial restoration of a p53 transcriptome, CB002 and other xanthine analogues impact on an S-phase cell cycle checkpoint (Hernandez Borrero et al., 2021). These small molecular weight compounds and others such as PG3-Oc and NSC59984 that restore p53 pathway responses merit further investigation as potential therapeutics in PDAC (Zhang et al., 2015; Prabhu et al., 2016; Zhang, 2017a; Zhang, 2017b; Zhang, 2018; Hernandez Borrero et al., 2021; Tian et al., 2021).

Mutations of the oncoprotein KRAS are very common in pancreatic cancer, and therefore there has always been a great deal of interest in targeting the MAPK pathway in the treatment of pancreatic cancer, but while there have been some promising pre-clinical results, KRAS inhibitors have shown to be relatively ineffective at treating pancreatic cancer in humans. Kinsey et al. established that inhibition of the MAPK pathway also leads to up-regulation of autophagy, which has been postulated as serving as a mechanism of drug resistance (Kinsey et al., 2019). Therefore, dual inhibition of the MAPK pathway and autophagy could theoretically lead to synergistic cell death. The combination proved synergistic in PDAC cell lines *in vitro* as well as in patient-derived xenografts grown in a murine model, as well as in melanoma and colorectal cancer models. Bryant et al. also examined the relationship between the MAPK pathway and autophagy and showed not only that dual inhibition of these pathways leads to increased cell death in PDAC cell lines, but also shed light on the mechanism of this synergy (Bryant et al., 2019) by showing that inhibition of two key members of the MAPK pathway—KRAS and ERK—lead to decreased metabolic functions, and would therefore lead to an increased dependence on autophagy to avoid cell death.

In summary, there is a breadth of literature examining the impact of autophagy on cancer initiation and growth. These studies have shown that the relationship between tumorigenesis and metastasis is complex, providing both pro- and anti-tumor effects. With this knowledge, various researchers have focused on both the inhibition and activation of autophagy. Harnessing the anti-tumor effect of autophagy inhibition has been attempted both *via* the use of existing drugs with broad mechanisms of action, such as chloroquine or hydroxychloroquine, as well as through the development of new targets to inhibit autophagy, such as VSP34 and ULK1 inhibitors. Likewise, other researchers have focused on promoters of autophagy, and have shown good effect with dual therapy with autophagy inhibitors. Lastly, it has been shown that dual targeting of the MAP kinase pathway and the autophagy pathway—especially in cancer with a high prevalence of KRAS mutation, such as pancreatic cancer—may result in increased tumor killing by inhibitors of the MAP kinase pathway by blocking autophagy, which could serve as a key mechanism of resistance.

**FIGURE 2:**
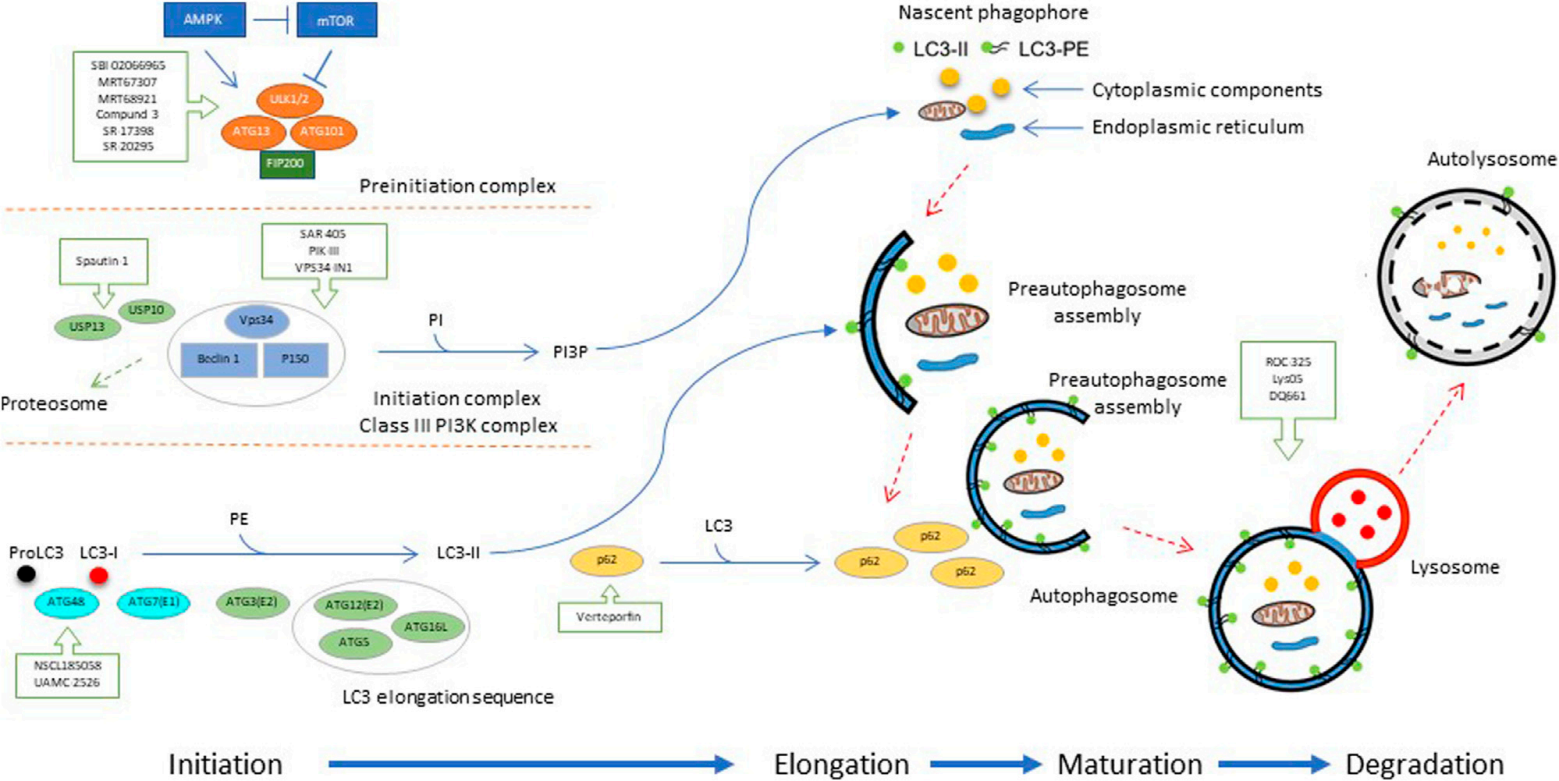
Modulation of multiple stages of autophagy process by small molecules. As illustrated in the schema, autophagy is a process where cells recycle proteins and other essential substrates and macromolecules including whole organelles such as mitochondria by forming an autophagosome. Autophagosomes confine and distribute their cargo for a highly regulated autophagy process which involves the fusion of autophagosomes with lysosomes. Therefore, each of the key complexes formed throughout the autophagy process involving preinitiation, initiation, elongation, maturation and degradation steps provide opportunities for therapeutic interventions by the small molecules that can modulate autophagic pathways. Under nutrient deprivation, mTOR is inactivated and AMPK is activated leading to phosphorylation of negative and positive regulatory sites on ULK1/2 within the preinitiation complex which subsequently activates the initiation complex or the class III PI-IIIK complex *via* phosphorylation of VPS34 and Beclin-1. The initiation complex involves the production of PI3P from the precursor PI needed for nucleation of the isolation of the autophagosome initiation membrane. Cellular concentrations of the initiation complex are regulated by a ubiquitination cascade which is regulated by USP10 and USP13 deubiquitination peptidases. Expansion of nascent precursor vesicles depends on the autophagosome LC3 protein which then conjugates with PE forming LC3-II protein which is derived from the LC3 elongation sequence of modifying enzymes. LC3-I is generated by proteolytic cleavage of proLC3 by the ATG4B. LC3-I is subsequently conjugated with lipids by a series of conjugating enzymes to form the LC3-II which then forms a stable complex with the membranes of autophagosomes. The p62 scaffold protein also plays an important role in the trafficking of proteins to the autophagosome by stably binding to the LC3-II protein. p62 also plays a role in apoptosis pathways. **Abbreviations:** mTOR, mammalian target of rapamycin; AMPK, 5′-AMP-activated protein kinase; ULK1/2, Unc-51-like autophagy activating kinase 1/2; VPS34, vacuolar protein sorting protein 34; PI3P, phosphatidylinositol-3-phosphate, PI: phosphatidylinositol, USP10 and USP13: deubiquitination peptidases, LC3: microtubule-associated protein 1A/1B light chain 3B, PE, phosphatidylethanolamine; LC3-II, conjugated form of the LC3 protein, ATG4B: protease autophagy-related protein 4B, p62, p62 is a receptor for cargo destined to be degraded by autophagy. Adapted from (Limpert et al., 2018).

A diagrammatic representation depicting modulation of the autophagy pathway by small molecules is shown in Figure 2. A list of compounds with activity as autophagy inhibitors is shown in Table 1.

**TABLE 1:** Selected compounds that modulate different phases of autophagy. Adapted from (Limpert et al., 2018).

Compound	Target	Novel features	Potency/Selectivity	Refs
SBI-0206965	ULK1 and ULK2	Selective inhibitor	ULK1: IC_50_ of 108 nM; ULK2: IC_50_ of 711 nM	Egan et al. (2015); Tang et al. (2017)
		Pyrimidine scaffold		
		Suppresses ULK1 downstream phosphorylation of VPS34 and Beclin-1		
		Induces apoptosis in NSCLC cells by destabilizing Bcl2 and Bclxl		
MRT67307	ULK1 and ULK2	*In vitro* inhibitor	ULK1: IC_50_ of 45 nM; ULK2: IC_50_ of 38 nM	Petherick et al. (2015)
		Pyrimidine scaffold		
		Also targets TBK1 and AMPK-related kinases		
MRT68921	ULK1 and ULK2	*In vitro* inhibitor	ULK1: IC_50_ of 2.9 nM; ULK2: IC_50_ of 1.1 nM	Petherick et al. (2015)
		Pyrimidine scaffold		
		Also targets TBK1 and AMPK-related kinases		
Compound 1	ULK1 and ULK2	Inhibitor	ULK1: IC_50_ of 5.3 nM; ULK2: IC_50_ of 13 nM; PDPK1: IC_50_ of 420 nM	Lazarus and Shokat, (2015)
		Pyrazole amino quinazoline scaffold		
		Crystal structure obtained with ULK1		
BX-795	PDK1	Inhibitor of PDK1	ULK1: IC_50_ of 87 nM; ULK2: IC_50_ of 310 nM; PDPK1: IC_50_ of 65 nM	Lazarus and Shokat, (2015)
		Also shown to inhibit ULK1, ULK2 and IKKε		
		Pyrimidine scaffold		
Compound 3	ULK1	Inhibitor	ULK1: IC_50_ of 120 nM; ULK2: IC_50_ of 360 nM; PDPK1: IC_50_ of 710 nM	Lazarus and Shokat, (2015)
		Pyrimidine scaffold		
		Crystal structure obtained with ULK1		
SR-17398	ULK1	Indazole-derived inhibitor	ULK1: IC_50_ of 22 μM	Wood et al. (2017)
		Mixture of four stereoisomers		
SR-20295	ULK1	Indazole-derived inhibitor	ULK1: IC_50_ of 45 nM *In vitro* microsome stability half-life of 225 min	Wood et al. (2017)
NSC185058	ATG4B	Inhibitor/antagonist	ATG4B IC_50_ of 51 μM	Akin et al. (2014); Huang et al. (2017)
		Targets autophagosome formation, and suppresses activation and lipidation of LC3		
UAMC-2526	ATG4B	Inhibitor	Plasma half-life of 126 min, and 70% metabolization after 30 min	Kurdi et al. (2017)
		Benzotropolone scaffold		
		Targets autophagosome formation		
		Inhibits starvation-induced autophagy *in vivo*		
SAR405	VPS34	Selective inhibitor	VPS34: IC_50_ of 1.2 nM and K_D_ of 1.5 nM	Ronan et al. (2014); Young et al. (2015); Hong et al. (2017)
		Tetrahydropyrimido-pyrimidinone scaffold		
		Dose-dependent inhibition		
		Targets autophagosome formation		
		Crystal structure obtained with VPS34		
PIK-III	VPS34	Selective and orally bioavailable inhibitor of VPS34	VPS34: IC_50_ of 18 nM; mTOR: IC_50_ of >9.1 μM	Dowdle et al. (2014); Honda et al. (2016)
		Pyrimidine scaffold		
		Inhibits autophagy and LC3 lipidation		
VPS34-IN1	VPS34	Selective cell-permeable inhibitor	VPS34: IC_50_ of 25 nM *in vitro*	Bago et al. (2014)
		Pyrimidine scaffold		
		Selectively inhibits class III PI3K		
Verteporfin	ATG	Concentration-dependent inhibition	CQ-verteporfin EGFP-LC3 cell IC_50_ of 1 μM Plasma concentrations after single intraperitoneal dose of 45 mg/kg: 122 μM at 2 h, 3.9 μM at 24 h	Donohue et al. (2011); Donohue et al. (2013); Donohue et al. (2014)
		Benzoporphyrin scaffold		
		Targets autophagosome formation and accumulation when co-treated with CQ		
		Targets p62: prevents autophagy-induced degradation of p62 in nutrient-deprived conditions		
Spautin-1	ATG	Autophagy inhibitor	Co-treatment with Spautin-1 improved imatinib mesylate-induced cytotoxicity of K562 leukemia cells: IC_50_ from 1.03 to 0.45 μM	Shao et al. (2014)
		Fluoroquinazoline scaffold		
		USP10 and USP13 inhibitor: promotes ubiquitination and decreases levels of Beclin-1		
		Targets autophagosome formation when co-treated with imatinib mesylate		
		Spautin-1 alone has no activity		
ROC-325	ATG	Orally bioavailable inhibitor	Acute myeloid leukemia cell IC_50_ range: 0.7–2.2 μM; A498 renal cell: IC_50_ of 4.9 μM	Nawrocki et al. (2016); Carew et al. (2017); Carew and Nawrocki, (2017)
		Chloroquinoline scaffold		
		Targets lysosomal function and autophagosome accumulation		
		∼10-fold more potent than HCQ		
		Exhibits significant anticancer activity against range of tumor types		
Lys05	ATG	Autophagy inhibitor	LN229 (glioma), 1205Lu (melanoma), c8161 (melanoma), HT-29 (colon) cell: IC_50_ range 4–8 μM	Amaravadi and Winkler, (2012); McAfee et al. (2012)
		Dimeric chloroquinoline scaffold		
		Targets lysosomal function		
DQ661	ATG	Inhibitor of autophagy and mTOR by targeting PPT1	Estimated A375P melanoma cell IC_50_ of ∼0.1 μM	Rebecca et al. (2017); Nicastri et al. (2018)
		Dimeric quinacrine scaffold		
		*In vivo* activity against melanoma, pancreatic cancer, and colorectal cancer tumor growth in mice		
		Can be used in combination with chemotherapy		

### Clinical Trials in PDAC

Various modulators of autophagy have been tested either alone or in combination with other agents in clinical trials for patients with PDAC. Chloroquine, and its less toxic derivative, hydroxychloroquine, are the among the best studied inhibitors of autophagy.

Hydroxychloroquine has been evaluated as a single agent in a phase II study published in 2014. In this study, 20 patients with previously treated metastatic PDAC received twice daily hydroxychloroquine, either 400 mg or 600 mg. Unfortunately, no patient demonstrated a response (Wolpin et al., 2014). In 2017, the results of a phase I trial combining chloroquine with standard of care gemcitabine were published. Although three out of nine enrolled patients had partial responses and a median overall survival (OS) of 7.6 months was reported, this did not outperform historical data with gemcitabine alone (Samaras et al., 2017). More recently, the results of a randomized phase II study of the combination of standard of care gemcitabine and nab-paclitaxel with or without hydroxychloroquine were published in 2019. In total, 112 patients with previously untreated metastatic or advanced PDAC were enrolled and were randomized 1:1. The primary endpoint was OS at 1 year. The addition of hydroxychloroquine resulted in a 12 months OS rate of 41% (95% CI, 27–53%) compared with 49% (95% CI, 35–61%) with chemotherapy alone. Furthermore, the authors reported no increase in progression free survival and there was a higher rate of toxicity, visual and gastrointestinal, in the hydroxychloroquine treatment group. Interestingly, the authors did report an improvement in overall response rate, 38.2% (n = 21) in the hydroxychloroquine group versus 21.1% (n = 12) in the non-hydroxychloroquine group, which was statically significant (*p* = 0.047) (Karasic et al., 2019).

Several studies have also investigated the role of autophagy promoting agents. The oral mTOR inhibitor everolimus has been studied in a phase II study in patients with gemcitabine-refractory metastatic pancreatic cancer. No complete or partial treatment responses were noted in this trial and the median progression-free survival and OS were 1.8 and 4.5 months, respectively. One patient (3%) had a biochemical response, defined as greater than or equal to 50% reduction in serum CA19-9 (Wolpin et al., 2009). Additional studies investigating single agent mTOR inhibitors have also been disappointing (Javle et al., 2010).

There are a number of ongoing clinical trials investigating novel autophagy-modulating agents and novel combinations of agents. For example, one trial is currently investigating newer combinations of chemotherapy (e.g., paclitaxel protein bound plus gemcitabine plus cisplatin) together with hydrochloroquine (NCT04669197). Hydroxychloroquine is also being combined with the vitamin D analogue, paricalcitol, and chemotherapy in a phase II trial (NCT04524702).

As discussed above, there is also interest in combining autophagy inhibitors with agents targeting the MAPK pathway. For example, two ongoing trials with two different MEK inhibitors, trametinib or binimetinib, combined with hydroxychloroquine are currently being tested in patients with PDAC (NCT03825289, NCT04132505). LY3214996, an ERK inhibitor, is currently being tested alone and in combination with hydroxychloroquine in a small phase two study (NCT04386057). The combination of the MEK inhibitor cobimetinib and hydroxychloroquine are also being tested in combination with immune checkpoint blockade in a phase I/II trial KRAS-mutated PDAC (NCT04214418).

A listing of clinical trials employing autophagy inhibitors is listed in Table 2.

**TABLE 2:** Clinical trials of autophagy inhibitors of pancreatic cancer. Source: clinicaltrials.gov.

Title	Status	Interventions	Url	NCT number
A phase I/II/Pharmacodynamic Study of Hydroxychloroquine in Combination With Gemcitabine/Abraxane to Inhibit Autophagy in Pancreatic Cancer	Active, not recruiting	Drug: Hydroxychloroquine (HCQ)|Drug: Gemcitabine|Drug: Abraxane	https://clinicaltrials.gov/ct2/show/NCT01506973	NCT01506973
LY3214996^+/−^HCQ in Pancreatic Cancer	Recruiting	Drug: Hydroxychloroquine Sulfate|Drug: LY3214996	https://clinicaltrials.gov/ct2/show/NCT04386057	NCT04386057
Binimetinib and Hydroxychloroquine in Treating Patients With KRAS Mutant Metastatic Pancreatic Cancer	Recruiting	Drug: binimetinib|Drug: Hydroxychloroquine	https://clinicaltrials.gov/ct2/show/NCT04132505	NCT04132505
Paricalcitol and Hydroxychloroquine in Combination With Gemcitabine and Nab-Paclitaxel for the Treatment of Advanced or Metastatic Pancreatic Cancer	Recruiting	Drug: Gemcitabine|Drug: Hydroxychloroquine|Drug: Nab-paclitaxel|Drug: Paricalcitol	https://clinicaltrials.gov/ct2/show/NCT04524702	NCT04524702
Randomized phase II Trial of Pre-Operative Gemcitabine and Nab Paclitacel With or With Out Hydroxychloroquine	Completed	Drug: gemcitabine|Drug: abraxane|Drug: hydroxychloroquine	https://clinicaltrials.gov/ct2/show/NCT01978184	NCT01978184
Short Course Radiation Therapy With Proton or Photon Beam Capecitabine and Hydroxychloroquine for Resectable Pancreatic Cancer	Active, not recruiting	Drug: Capecitabine| Drug: Hydroxychloroquine|Radiation: Proton or Photon Radiation Therapy	https://clinicaltrials.gov/ct2/show/NCT01494155	NCT01494155
Study of Combination Therapy With the MEK Inhibitor, cobimetinib, Immune Checkpoint Blockade, atezolizumab, and the AUTOphagy Inhibitor, Hydroxychloroquine in KRAS-mutated Advanced Malignancies	Recruiting	Drug: cobimetinib| Drug: Hydroxychloroquine|Drug: atezolizumab| Drug: Hydroxychloroquine| Drug: atezolizumab	https://clinicaltrials.gov/ct2/show/NCT04214418	NCT04214418
Trametinib and Hydroxychloroquine in Treating Patients With Pancreatic Cancer	Recruiting	Drug: Hydroxychloroquine|Drug: trametinib	https://clinicaltrials.gov/ct2/show/NCT03825289	NCT03825289
Phase II Study of Paclitaxel Protein Bound + Gemcitabine + Cisplatin + Hydrochloroquine as Treatment in Untreated Pancreas Cancer	Recruiting	Drug: Paclitaxel protein bound|Drug: Gemcitabine|Drug: Cisplatin|Drug: Hydroxychloroquine	https://clinicaltrials.gov/ct2/show/NCT04669197	NCT04669197

## Discussion

Recent advances in our understanding of autophagy and evidence suggesting that it may be necessary for PDAC tumorigenesis, maintenance, and metastasis has rekindled enthusiasm to target this process for therapeutic benefit. Development of effective therapies has been slow, in part due to the tremendous complexity and dynamic roles autophagy plays in both cell survival and cell death. No agent to date has demonstrated clear clinical benefit but ongoing trials will hopefully shed light on biological effects and emerging resistance pathways.

Traditionally, autophagy has been described as an adaptive mechanism through which cells facing stress or starvation are able to maintain viability. The role of autophagy in tumorigenesis is less clear, but we do know that established PDAC tumors rely on chronically elevated levels of basal autophagy. Furthermore, there is evidence that autophagy may also be required for metastasis. The unique PDAC tumor immune microenvironment represents a hypoxic, acidic, nutrient-poor setting in which autophagy has been repeatedly demonstrated to be upregulated. Adding further complexity is the fact that autophagy also plays a role in immune cell function and therefore, it is possible that modulating this process may impact immune response to cancer.

Several potential predictive biomarkers, such as ATG5 and LC3-II, are currently being studied and may help to ensure adequate dosing of autophagy targeting agents. Incorporation of biomarker studies into future clinical trials will be necessary to confirm utility.

With the identification of novel autophagy pathway components and the development of more specific pharmacologic agents, future trials will likely hold more promise. Recent preclinical data supporting combinatory therapy with MAPK pathway and autophagy inhibition with chloroquine has led to the activation of multiple clinical trials with these agents. Additional novel agents with preclinical activity such as ONC212, with the ability to inhibit autophagy, may be well-suited for further study in combination with MEK inhibitors or other agents in pancreatic cancer. Therapeutics targeting other molecular drivers in PDAC, such as mutant p53, may have future use in this disease. Further investigation with improved preclinical models and biomarker directed clinical trials is warranted to further our understanding of autophagy modulation and ultimately improve outcomes in PDAC.

## Data Availability

The original contributions presented in the study are included in the article/Supplementary Material, further inquiries can be directed to the corresponding authors.

## References

[B1] Akin D. Wang S. K. Habibzadegah-Tari P. Law B. Ostrov D. Li M. (2014). A Novel ATG4B Antagonist Inhibits Autophagy and Has a Negative Impact on Osteosarcoma Tumors. Autophagy 10 (11), 2021–2035. 10.4161/auto.32229 25483883 PMC4502682

[B2] Allen J. E. Krigsfeld G. Mayes P. A. Patel L. Dicker D. T. Patel A. S. (2013). Dual Inactivation of Akt and ERK by TIC10 Signals Foxo3a Nuclear Translocation, TRAIL Gene Induction, and Potent Antitumor Effects. Sci. Transl. Med. 5 (171), 171ra17. 10.1126/scitranslmed.3004828 PMC4535715 23390247

[B3] Amaravadi R. K. Winkler J. D. (2012). Lys05: a New Lysosomal Autophagy Inhibitor. Autophagy 8 (9), 1383–1384. 10.4161/auto.20958 22878685 PMC3442884

[B4] B'Chir W. Maurin A. C. Carraro V. Averous J. Jousse C. Muranishi Y. (2013). The eIF2α/ATF4 Pathway Is Essential for Stress-Induced Autophagy Gene Expression. Nucleic Acids Res. 41 (16), 7683–7699. 10.1093/nar/gkt563 23804767 PMC3763548

[B5] Bago R. Malik N. Munson M. J. Prescott A. R. Davies P. Sommer E. (2014). Characterization of VPS34-IN1, a Selective Inhibitor of Vps34, Reveals that the Phosphatidylinositol 3-Phosphate-Binding SGK3 Protein Kinase Is a Downstream Target of Class III Phosphoinositide 3-kinase. Biochem. J. 463 (3), 413–427. 10.1042/BJ20140889 25177796 PMC4209782

[B6] Bassett E. A. Wang W. Rastinejad F. El-Deiry W. S. (2008). Structural and Functional Basis for Therapeutic Modulation of P53 Signaling. Clin. Cancer Res. 14 (20), 6376–6386. 10.1158/1078-0432.CCR-08-1526 18927276

[B7] Bellot G. Garcia-Medina R. Gounon P. Chiche J. Roux D. Pouysségur J. (2009). Hypoxia-induced Autophagy Is Mediated through Hypoxia-Inducible Factor Induction of BNIP3 and BNIP3L via Their BH3 Domains. Mol. Cel. Biol. 29 (10), 2570–2581. 10.1128/MCB.00166-09 PMC2682037 19273585

[B8] Bian J. Almhanna K. (2021). Pancreatic Cancer and Immune Checkpoint Inhibitors-Still a Long Way to Go. Transl. Gastroenterol. Hepatol. 6, 6. 10.21037/tgh.2020.04.03 33409400 PMC7724172

[B9] Bryant K. L. Stalnecker C. A. Zeitouni D. Klomp J. E. Peng S. Tikunov A. P. (2019). Combination of ERK and Autophagy Inhibition as a Treatment Approach for Pancreatic Cancer. Nat. Med. 25 (4), 628–640. 10.1038/s41591-019-0368-8 30833752 PMC6484853

[B10] Carew J. S. Espitia C. M. Zhao W. Han Y. Visconte V. Phillips J. (2017). Disruption of Autophagic Degradation with ROC-325 Antagonizes Renal Cell Carcinoma Pathogenesis. Clin. Cancer Res. 23 (11), 2869–2879. 10.1158/1078-0432.CCR-16-1742 27881580 PMC5593077

[B11] Carew J. S. Nawrocki S. T. (2017). Drain the Lysosome: Development of the Novel Orally Available Autophagy Inhibitor ROC-325. Autophagy 13 (4), 765–766. 10.1080/15548627.2017.1280222 28118053 PMC5388230

[B12] Carew J. S. Nawrocki S. T. Kahue C. N. Zhang H. Yang C. Chung L. (2007). Targeting Autophagy Augments the Anticancer Activity of the Histone Deacetylase Inhibitor SAHA to Overcome Bcr-Abl-Mediated Drug Resistance. Blood 110 (1), 313–322. 10.1182/blood-2006-10-050260 17363733 PMC1896119

[B13] Carneiro B. A. El-Deiry W. S. (2020). Targeting Apoptosis in Cancer Therapy. Nat. Rev. Clin. Oncol. 17 (7), 395–417. 10.1038/s41571-020-0341-y 32203277 PMC8211386

[B14] Chen S. Sang N. (2016). Hypoxia-Inducible Factor-1: A Critical Player in the Survival Strategy of Stressed Cells. J. Cel. Biochem. 117 (2), 267–278. 10.1002/jcb.25283 PMC4715696 26206147

[B15] Clarke A. J. Simon A. K. (2019). Autophagy in the Renewal, Differentiation and Homeostasis of Immune Cells. Nat. Rev. Immunol. 19 (3), 170–183. 10.1038/s41577-018-0095-2 30531943

[B16] Cunha L. D. Yang M. Carter R. Guy C. Harris L. Crawford J. C. (2018). LC3-Associated Phagocytosis in Myeloid Cells Promotes Tumor Immune Tolerance. Cell 175 (2), 429–e16. 10.1016/j.cell.2018.08.061 30245008 PMC6201245

[B17] Deegan S. Koryga I. Glynn S. A. Gupta S. Gorman A. M. Samali A. (2015). A Close Connection between the PERK and IRE Arms of the UPR and the Transcriptional Regulation of Autophagy. Biochem. Biophys. Res. Commun. 456 (1), 305–311. 10.1016/j.bbrc.2014.11.076 25475719

[B18] Deegan S. Saveljeva S. Gorman A. M. Samali A. (2013). Stress-induced Self-Cannibalism: on the Regulation of Autophagy by Endoplasmic Reticulum Stress. Cell. Mol. Life Sci. 70 (14), 2425–2441. 10.1007/s00018-012-1173-4 23052213 PMC11113399

[B19] Dennis M. D. McGhee N. K. Jefferson L. S. Kimball S. R. (2013). Regulated in DNA Damage and Development 1 (REDD1) Promotes Cell Survival during Serum Deprivation by Sustaining Repression of Signaling through the Mechanistic Target of Rapamycin in Complex 1 (mTORC1). Cell. Signal. 25 (12), 2709–2716. 10.1016/j.cellsig.2013.08.038 24018049 PMC3867791

[B20] Dey S. Baird T. D. Zhou D. Palam L. R. Spandau D. F. Wek R. C. (2010). Both transcriptional Regulation and Translational Control of ATF4 Are central to the Integrated Stress Response. J. Biol. Chem. 285 (43), 33165–33174. 10.1074/jbc.M110.167213 20732869 PMC2963398

[B21] Dolgin E. (2019). Anticancer Autophagy Inhibitors Attract ‘resurgent' Interest. Nat. Rev. Drug Discov. 18 (6), 408–410. 10.1038/d41573-019-00072-1 31160763

[B22] Donohue E. Balgi A. D. Komatsu M. Roberge M. (2014). Induction of Covalently Crosslinked P62 Oligomers with Reduced Binding to Polyubiquitinated Proteins by the Autophagy Inhibitor Verteporfin. PLoS One 9, e114964. 10.1371/journal.pone.0114964 25494214 PMC4262463

[B23] Donohue E. Thomas A. Maurer N. Manisali I. Zeisser-Labouebe M. Zisman N. (2013). The Autophagy Inhibitor Verteporfin Moderately Enhances the Antitumor Activity of Gemcitabine in a Pancreatic Ductal Adenocarcinoma Model. J. Cancer 4 (7), 585–596. 10.7150/jca.7030 24069069 PMC3781989

[B24] Donohue E. Tovey A. Vogl A. W. Arns S. Sternberg E. Young R. N. (2011). Inhibition of Autophagosome Formation by the Benzoporphyrin Derivative Verteporfin. J. Biol. Chem. 286 (9), 7290–7300. 10.1074/jbc.M110.139915 21193398 PMC3044985

[B25] Dowdle W. E. Nyfeler B. Nagel J. Elling R. A. Liu S. Triantafellow E. (2014). Selective VPS34 Inhibitor Blocks Autophagy and Uncovers a Role for NCOA4 in Ferritin Degradation and Iron Homeostasis *In Vivo* . Nat. Cel. Biol. 16 (11), 1069–1079. 10.1038/ncb3053 25327288

[B26] Egan D. F. Chun M. G. Vamos M. Zou H. Rong J. Miller C. J. (2015). Small Molecule Inhibition of the Autophagy Kinase ULK1 and Identification of ULK1 Substrates. Mol. Cel 59 (2), 285–297. 10.1016/j.molcel.2015.05.031 PMC4530630 26118643

[B27] Ferrarini I. (2021). ONC212 Is a Novel Mitocan Acting Synergistically with Glycolysis Inhibition in Pancreatic Cancer. Mol. Cancer Ther.. p. molcanther.MCT-20-0962-A.2020. 10.1158/1535-7163.MCT-20-0962 PMC8419089 34224362

[B28] Firat E. Weyerbrock A. Gaedicke S. Grosu A. L. Niedermann G. (2012). Chloroquine or Chloroquine-PI3K/Akt Pathway Inhibitor Combinations Strongly Promote γ-irradiation-induced Cell Death in Primary Stem-like Glioma Cells. PLoS One 7, e47357. 10.1371/journal.pone.0047357 23091617 PMC3473017

[B29] Frieboes H. B. Huang J. S. Yin W. C. McNally L. R. (2014). Chloroquine-mediated Cell Death in Metastatic Pancreatic Adenocarcinoma through Inhibition of Autophagy. Jop 15 (2), 189–197. 10.6092/1590-8577/1900 24618445

[B30] Fujii S. Mitsunaga S. Yamazaki M. Hasebe T. Ishii G. Kojima M. (2008). Autophagy Is Activated in Pancreatic Cancer Cells and Correlates with Poor Patient Outcome. Cancer Sci. 99 (9), 1813–1819. 10.1111/j.1349-7006.2008.00893.x 18616529 PMC11159933

[B31] Germic N. Frangez Z. Yousefi S. Simon H. U. (2019). Regulation of the Innate Immune System by Autophagy: Monocytes, Macrophages, Dendritic Cells and Antigen Presentation. Cell Death Differ. 26 (4), 715–727. 10.1038/s41418-019-0297-6 30737475 PMC6460400

[B32] Ghadimi M. P. Lopez G. Torres K. E. Belousov R. Young E. D. Liu J. (2012). Targeting the PI3K/mTOR axis, Alone and in Combination with Autophagy Blockade, for the Treatment of Malignant Peripheral Nerve Sheath Tumors. Mol. Cancer Ther. 11 (8), 1758–1769. 10.1158/1535-7163.MCT-12-0015 22848094 PMC3416967

[B33] Golan T. Hammel P. Reni M. Van Cutsem E. Macarulla T. Hall M. J. (2019). Maintenance Olaparib for Germline BRCA-Mutated Metastatic Pancreatic Cancer. N. Engl. J. Med. 381 (4), 317–327. 10.1056/NEJMoa1903387 31157963 PMC6810605

[B34] Görgülü K. Diakopoulos K. N. Ai J. Schoeps B. Kabacaoglu D. Karpathaki A. F. (2019). Levels of the Autophagy-Related 5 Protein Affect Progression and Metastasis of Pancreatic Tumors in Mice. Gastroenterology 156 (1), 203–e20. 10.1053/j.gastro.2018.09.053 30296435

[B35] Guan B. J. Krokowski D. Majumder M. Schmotzer C. L. Kimball S. R. Merrick W. C. (2014). Translational Control during Endoplasmic Reticulum Stress beyond Phosphorylation of the Translation Initiation Factor eIF2α. J. Biol. Chem. 289 (18), 12593–12611. 10.1074/jbc.M113.543215 24648524 PMC4007450

[B36] Guo J. Y. Karsli-Uzunbas G. Mathew R. Aisner S. C. Kamphorst J. J. Strohecker A. M. (2013). Autophagy Suppresses Progression of K-Ras-Induced Lung Tumors to Oncocytomas and Maintains Lipid Homeostasis. Genes Dev. 27 (13), 1447–1461. 10.1101/gad.219642.113 23824538 PMC3713426

[B37] Guo J. Y. Teng X. Laddha S. V. Ma S. Van Nostrand S. C. Yang Y. (2016). Autophagy Provides Metabolic Substrates to Maintain Energy Charge and Nucleotide Pools in Ras-Driven Lung Cancer Cells. Genes Dev. 30 (15), 1704–1717. 10.1101/gad.283416.116 27516533 PMC5002976

[B38] Han W. Pan H. Chen Y. Sun J. Wang Y. Li J. (2011). EGFR Tyrosine Kinase Inhibitors Activate Autophagy as a Cytoprotective Response in Human Lung Cancer Cells. PLoS One 6, e18691. 10.1371/journal.pone.0018691 21655094 PMC3107207

[B39] Harding H. P. Zhang Y. Bertolotti A. Zeng H. Ron D. (2000). Perk Is Essential for Translational Regulation and Cell Survival during the Unfolded Protein Response. Mol. Cel. 5 (5), 897–904. 10.1016/s1097-2765(00)80330-5 10882126

[B40] Hernandez Borrero L. Dicker D. T. Santiago J. Sanders J. Tian X. Ahsan N. (2021). A Subset of CB002 Xanthine Analogs Bypass P53-Signaling to Restore a P53 Transcriptome and Target an S-phase Cell Cycle Checkpoint in Tumors with Mutated-P53. Elife 10, 10. 10.7554/eLife.70429 PMC8321552 34324416

[B41] Hernández Borrero L. J. El-Deiry W. S. (2021). Tumor Suppressor P53: Biology, Signaling Pathways, and Therapeutic Targeting. Biochim. Biophys. Acta (Bba) - Rev. Cancer 1876 (1), 188556. 10.1016/j.bbcan.2021.188556 PMC8730328 33932560

[B42] Hernandez-Borrero L. J. Zhang S. Lulla A. Dicker D. T. El-Deiry W. S. (2018). CB002, a Novel P53 Tumor Suppressor Pathway-Restoring Small Molecule Induces Tumor Cell Death through the Pro-apoptotic Protein NOXA. Cell Cycle 17 (5), 557–567. 10.1080/15384101.2017.1346762 28749203 PMC5969548

[B43] Honda A. Harrington E. Cornella-Taracido I. Furet P. Knapp M. S. Glick M. (2016). Potent, Selective, and Orally Bioavailable Inhibitors of VPS34 Provide Chemical Tools to Modulate Autophagy *In Vivo* . ACS Med. Chem. Lett. 7 (1), 72–76. 10.1021/acsmedchemlett.5b00335 26819669 PMC4716600

[B44] Hong Z. Pedersen N. M. Wang L. Torgersen M. L. Stenmark H. Raiborg C. (2017). PtdIns3P Controls mTORC1 Signaling through Lysosomal Positioning. J. Cel. Biol. 216 (12), 4217–4233. 10.1083/jcb.201611073 PMC5716264 29030394

[B45] Horsman M. R. Overgaard J. (2016). The Impact of Hypoxia and its Modification of the Outcome of Radiotherapy. J. Radiat. Res. 57 (Suppl. 1), i90. 10.1093/jrr/rrw007 26983987 PMC4990104

[B46] Hu Y. L. Jahangiri A. Delay M. Aghi M. K. (2012). Tumor Cell Autophagy as an Adaptive Response Mediating Resistance to Treatments Such as Antiangiogenic Therapy. Cancer Res. 72 (17), 4294–4299. 10.1158/0008-5472.CAN-12-1076 22915758 PMC3432684

[B47] Huang T. Kim C. K. Alvarez A. A. Pangeni R. P. Wan X. Song X. (2017). MST4 Phosphorylation of ATG4B Regulates Autophagic Activity, Tumorigenicity, and Radioresistance in Glioblastoma. Cancer Cell 32 (6), 840–e8. 10.1016/j.ccell.2017.11.005 29232556 PMC5734934

[B48] Jacquel A. Obba S. Boyer L. Dufies M. Robert G. Gounon P. (2012). Autophagy Is Required for CSF-1-Induced Macrophagic Differentiation and Acquisition of Phagocytic Functions. Blood 119 (19), 4527–4531. 10.1182/blood-2011-11-392167 22452982

[B49] Javle M. M. Shroff R. T. Xiong H. Varadhachary G. A. Fogelman D. Reddy S. A. (2010). Inhibition of the Mammalian Target of Rapamycin (mTOR) in Advanced Pancreatic Cancer: Results of Two Phase II Studies. BMC Cancer 10, 368. 10.1186/1471-2407-10-368 20630061 PMC2910694

[B50] Jhaveri A. V. (2020). Abstract 6225: Addition of TRAIL Receptor Agonists after Treatment with ONC201 or ONC212 Converts Pancreatic Cancer Cells from Anti-proliferative to Apoptotic *In Vitro* . Cancer Res. 80 (16 Suppl. ment), 6225.

[B51] Kaelin W. G. Jr. (2011). Cancer and Altered Metabolism: Potential Importance of Hypoxia-Inducible Factor and 2-oxoglutarate-dependent Dioxygenases. Cold Spring Harb. Symp. Quant. Biol. 76, 335–345. 10.1101/sqb.2011.76.010975 22089927 PMC4197849

[B52] Kang R. Tang D. Schapiro N. E. Livesey K. M. Farkas A. Loughran P. (2010). The Receptor for Advanced Glycation End Products (RAGE) Sustains Autophagy and Limits Apoptosis, Promoting Pancreatic Tumor Cell Survival. Cel. Death Differ. 17 (4), 666–676. 10.1038/cdd.2009.149 PMC3417122 19834494

[B53] Karasic T. B. O'Hara M. H. Loaiza-Bonilla A. Reiss K. A. Teitelbaum U. R. Borazanci E. (2019). Effect of Gemcitabine and Nab-Paclitaxel with or without Hydroxychloroquine on Patients with Advanced Pancreatic Cancer: A Phase 2 Randomized Clinical Trial. JAMA Oncol. 5 (7), 993–998. 10.1001/jamaoncol.2019.0684 31120501 PMC6547080

[B54] Karsli-Uzunbas G. Guo J. Y. Price S. Teng X. Laddha S. V. Khor S. (2014). Autophagy Is Required for Glucose Homeostasis and Lung Tumor Maintenance. Cancer Discov. 4 (8), 914–927. 10.1158/2159-8290.CD-14-0363 24875857 PMC4125614

[B55] Kazemi S. Mounir Z. Baltzis D. Raven J. F. Wang S. Krishnamoorthy J. L. (2007). A Novel Function of eIF2alpha Kinases as Inducers of the Phosphoinositide-3 Kinase Signaling Pathway. Mol. Biol. Cel. 18 (9), 3635–3644. 10.1091/mbc.e07-01-0053 PMC1951772 17596516

[B56] Kim J. Kundu M. Viollet B. Guan K. L. (2011). AMPK and mTOR Regulate Autophagy through Direct Phosphorylation of Ulk1. Nat. Cel. Biol. 13 (2), 132–141. 10.1038/ncb2152 PMC3987946 21258367

[B57] Kinsey C. G. Camolotto S. A. Boespflug A. M. Guillen K. P. Foth M. Truong A. (2019). Protective Autophagy Elicited by RAF→MEK→ERK Inhibition Suggests a Treatment Strategy for RAS-Driven Cancers. Nat. Med. 25 (4), 620–627. 10.1038/s41591-019-0367-9 30833748 PMC6452642

[B58] Kline C. L. Van den Heuvel A. P. Allen J. E. Prabhu V. V. Dicker D. T. El-Deiry W. S. (2016). ONC201 Kills Solid Tumor Cells by Triggering an Integrated Stress Response Dependent on ATF4 Activation by Specific eIF2α Kinases. Sci. Signal. 9, ra18. 10.1126/scisignal.aac4374 26884600 PMC4968406

[B59] Klionsky D. J. Abeliovich H. Agostinis P. Agrawal D. K. Aliev G. Askew D. S. (2008). Guidelines for the Use and Interpretation of Assays for Monitoring Autophagy in Higher Eukaryotes. Autophagy 4 (2), 151–175. 10.4161/auto.5338 18188003 PMC2654259

[B60] Kouroku Y. Fujita E. Tanida I. Ueno T. Isoai A. Kumagai H. (2007). ER Stress (PERK/eIF2alpha Phosphorylation) Mediates the Polyglutamine-Induced LC3 Conversion, an Essential Step for Autophagy Formation. Cel. Death Differ. 14 (2), 230–239. 10.1038/sj.cdd.4401984 16794605

[B61] Kroemer G. Mariño G. Levine B. (2010). Autophagy and the Integrated Stress Response. Mol. Cel. 40 (2), 280–293. 10.1016/j.molcel.2010.09.023 PMC3127250 20965422

[B62] Kume S. Uzu T. Horiike K. Chin-Kanasaki M. Isshiki K. Araki S. (2010). Calorie Restriction Enhances Cell Adaptation to Hypoxia through Sirt1-dependent Mitochondrial Autophagy in Mouse Aged Kidney. J. Clin. Invest. 120 (4), 1043–1055. 10.1172/JCI41376 20335657 PMC2846062

[B63] Kurdi A. Cleenewerck M. Vangestel C. Lyssens S. Declercq W. Timmermans J. P. (2017). ATG4B Inhibitors with a Benzotropolone Core Structure Block Autophagy and Augment Efficiency of Chemotherapy in Mice. Biochem. Pharmacol. 138, 150–162. 10.1016/j.bcp.2017.06.119 28642033

[B64] Kuribayashi K. Krigsfeld G. Wang W. Xu J. Mayes P. A. Dicker D. T. (2008). TNFSF10 (TRAIL), a P53 Target Gene that Mediates P53-dependent Cell Death. Cancer Biol. Ther. 7 (12), 2034–2038. 10.4161/cbt.7.12.7460 19106633

[B65] Lazarus M. B. Novotny C. J. Shokat K. M. (2015). Structure of the Human Autophagy Initiating Kinase ULK1 in Complex with Potent Inhibitors. ACS Chem. Biol. 10 (1), 257–261. 10.1021/cb500835z 25551253 PMC4301081

[B66] Lazarus M. B. Shokat K. M. (2015). Discovery and Structure of a New Inhibitor Scaffold of the Autophagy Initiating Kinase ULK1. Bioorg. Med. Chem. 23 (17), 5483–5488. 10.1016/j.bmc.2015.07.034 26275681 PMC4864979

[B67] Lebovitz C. B. Robertson A. G. Goya R. Jones S. J. Morin R. D. Marra M. A. (2015). Cross-cancer Profiling of Molecular Alterations within the Human Autophagy Interaction Network. Autophagy 11 (9), 1668–1687. 10.1080/15548627.2015.1067362 26208877 PMC4590660

[B68] Lev A. Lulla A. R. Wagner J. Ralff M. D. Kiehl J. B. Zhou Y. (2017). Anti-pancreatic Cancer Activity of ONC212 Involves the Unfolded Protein Response (UPR) and Is Reduced by IGF1-R and GRP78/BIP. Oncotarget 8 (47), 81776–81793. 10.18632/oncotarget.20819 29137221 PMC5669847

[B69] Levine B. Kroemer G. (2008). Autophagy in the Pathogenesis of Disease. Cell 132 (1), 27–42. 10.1016/j.cell.2007.12.018 18191218 PMC2696814

[B70] Li Y. Hahn T. Garrison K. Cui Z. H. Thorburn A. Thorburn J. (2012). The Vitamin E Analogue α-TEA Stimulates Tumor Autophagy and Enhances Antigen Cross-Presentation. Cancer Res. 72 (14), 3535–3545. 10.1158/0008-5472.CAN-11-3103 22745370 PMC3576035

[B71] Liang X. De Vera M. E. Buchser W. J. Romo de Vivar Chavez A. Loughran P. Beer Stolz D. (2012). Inhibiting Systemic Autophagy during Interleukin 2 Immunotherapy Promotes Long-Term Tumor Regression. Cancer Res. 72 (11), 2791–2801. 10.1158/0008-5472.CAN-12-0320 22472122 PMC3417121

[B72] Liberti M. V. Locasale J. W. (2016). The Warburg Effect: How Does it Benefit Cancer Cells. Trends Biochem. Sci. 41 (3), 211–218. 10.1016/j.tibs.2015.12.001 26778478 PMC4783224

[B73] Limpert A. S. Lambert L. J. Bakas N. A. Bata N. Brun S. N. Shaw R. J. (2018). Autophagy in Cancer: Regulation by Small Molecules. Trends Pharmacol. Sci. 39 (12), 1021–1032. 10.1016/j.tips.2018.10.004 30454769 PMC6349222

[B74] Lin J. H. Li H. Yasumura D. Cohen H. R. Zhang C. Panning B. (2007). IRE1 Signaling Affects Cell Fate during the Unfolded Protein Response. Science 318 (5852), 944–949. 10.1126/science.1146361 17991856 PMC3670588

[B75] Lin J. H. Li H. Zhang Y. Ron D. Walter P. (2009). Divergent Effects of PERK and IRE1 Signaling on Cell Viability. PLoS One 4, e4170. 10.1371/journal.pone.0004170 19137072 PMC2614882

[B76] Liu C. Y. Schröder M. Kaufman R. J. (2000). Ligand-independent Dimerization Activates the Stress Response Kinases IRE1 and PERK in the Lumen of the Endoplasmic Reticulum. J. Biol. Chem. 275 (32), 24881–24885. 10.1074/jbc.M004454200 10835430

[B77] Maes H. Kuchnio A. Peric A. Moens S. Nys K. De Bock K. (2014). Tumor Vessel Normalization by Chloroquine Independent of Autophagy. Cancer Cell 26 (2), 190–206. 10.1016/j.ccr.2014.06.025 25117709

[B78] Majmundar A. J. Wong W. J. Simon M. C. (2010). Hypoxia-inducible Factors and the Response to Hypoxic Stress. Mol. Cel. 40 (2), 294–309. 10.1016/j.molcel.2010.09.022 PMC3143508 20965423

[B79] Masson N. Ratcliffe P. J. (2014). Hypoxia Signaling Pathways in Cancer Metabolism: the Importance of Co-selecting Interconnected Physiological Pathways. Cancer Metab. 2 (1), 3. 10.1186/2049-3002-2-3 24491179 PMC3938304

[B80] Mazure N. M. Pouysségur J. (2010). Hypoxia-induced Autophagy: Cell Death or Cell Survival. Curr. Opin. Cel. Biol. 22 (2), 177–180. 10.1016/j.ceb.2009.11.015 20022734

[B81] McAfee Q. Zhang Z. Samanta A. Levi S. M. Ma X. H. Piao S. (2012). Autophagy Inhibitor Lys05 Has Single-Agent Antitumor Activity and Reproduces the Phenotype of a Genetic Autophagy Deficiency. Proc. Natl. Acad. Sci. U S A. 109 (21), 8253–8258. 10.1073/pnas.1118193109 22566612 PMC3361415

[B82] Miller K. D. Siegel R. L. Lin C. C. Mariotto A. B. Kramer J. L. Rowland J. H. (2016). Cancer Treatment and Survivorship Statistics, 2016. CA Cancer J. Clin. 66 (1), 271–289. 10.3322/caac.21349 27253694

[B83] Minassian L. M. Cotechini T. Huitema E. Graham C. H. (2019). Hypoxia-Induced Resistance to Chemotherapy in Cancer. Adv. Exp. Med. Biol. 1136, 123–139. 10.1007/978-3-030-12734-3_9 31201721

[B84] Mizushima N. Yoshimori T. Ohsumi Y. (2011). The Role of Atg Proteins in Autophagosome Formation. Annu. Rev. Cel. Dev. Biol. 27, 107–132. 10.1146/annurev-cellbio-092910-154005 21801009

[B85] Moore M. J. Goldstein D. Hamm J. Figer A. Hecht J. R. Gallinger S. (2007). Erlotinib Plus Gemcitabine Compared with Gemcitabine Alone in Patients with Advanced Pancreatic Cancer: A Phase III Trial of the National Cancer Institute of Canada Clinical Trials Group. J. Clin. Oncol. 25 (15), 1960–1966. 10.1200/JCO.2006.07.9525 17452677

[B86] Mortensen M. Soilleux E. J. Djordjevic G. Tripp R. Lutteropp M. Sadighi-Akha E. (2011). The Autophagy Protein Atg7 Is Essential for Hematopoietic Stem Cell Maintenance. J. Exp. Med. 208 (3), 455–467. 10.1084/jem.20101145 21339326 PMC3058574

[B87] Mulcahy Levy J. M. Thorburn A. (2020). Autophagy in Cancer: Moving from Understanding Mechanism to Improving Therapy Responses in Patients. Cel. Death Differ. 27 (3), 843–857. 10.1038/s41418-019-0474-7 PMC7206017 31836831

[B88] Nawrocki S. T. Han Y. Visconte V. Phillips J. G. Przychodzen B. P. Maciejewski J. P. (2016). Development of ROC-325: A Novel Small Molecule Inhibitor of Autophagy with Promising Anti-leukemic Activity. 128 (22), p. 525. 10.1182/blood.v128.22.525.525

[B89] Nicastri M. C. Rebecca V. W. Amaravadi R. K. Winkler J. D. (2018). Dimeric Quinacrines as Chemical Tools to Identify PPT1, a New Regulator of Autophagy in Cancer Cells. Mol. Cel. Oncol. 5, e1395504. 10.1080/23723556.2017.1395504 PMC5791860 29404393

[B90] Novak I. Kirkin V. McEwan D. G. Zhang J. Wild P. Rozenknop A. (2010). Nix Is a Selective Autophagy Receptor for Mitochondrial Clearance. EMBO Rep. 11 (1), 45–51. 10.1038/embor.2009.256 20010802 PMC2816619

[B91] Pakos-Zebrucka K. Koryga I. Mnich K. Ljujic M. Samali A. Gorman A. M. (2016). The Integrated Stress Response. EMBO Rep. 17 (10), 1374–1395. 10.15252/embr.201642195 27629041 PMC5048378

[B92] Perera R. M. Stoykova S. Nicolay B. N. Ross K. N. Fitamant J. Boukhali M. (2015). Transcriptional Control of Autophagy-Lysosome Function Drives Pancreatic Cancer Metabolism. Nature 524 (7565), 361–365. 10.1038/nature14587 26168401 PMC5086585

[B93] Petherick K. J. Conway O. J. Mpamhanga C. Osborne S. A. Kamal A. Saxty B. (2015). Pharmacological Inhibition of ULK1 Kinase Blocks Mammalian Target of Rapamycin (mTOR)-dependent Autophagy. J. Biol. Chem. 290 (48), 28726. 10.1074/jbc.A114.627778 26614783 PMC4661389

[B94] Pietrocola F. Pol J. Vacchelli E. Rao S. Enot D. P. Baracco E. E. (2016). Caloric Restriction Mimetics Enhance Anticancer Immunosurveillance. Cancer Cell 30 (1), 147–160. 10.1016/j.ccell.2016.05.016 27411589 PMC5715805

[B95] Piffoux M. Eriau E. Cassier P. A. (2021). Autophagy as a Therapeutic Target in Pancreatic Cancer. Br. J. Cancer 124 (2), 333–344. 10.1038/s41416-020-01039-5 32929194 PMC7852577

[B96] Prabhu V. V. Allen J. E. Dicker D. T. El-Deiry W. S. (2015). Small-Molecule ONC201/TIC10 Targets Chemotherapy-Resistant Colorectal Cancer Stem-like Cells in an Akt/Foxo3a/TRAIL-dependent Manner. Cancer Res. 75 (7), 1423–1432. 10.1158/0008-5472.CAN-13-3451 25712124 PMC4537643

[B97] Prabhu V. V. Hong B. Allen J. E. Zhang S. Lulla A. R. Dicker D. T. (2016). Small-Molecule Prodigiosin Restores P53 Tumor Suppressor Activity in Chemoresistant Colorectal Cancer Stem Cells via C-Jun-Mediated ΔNp73 Inhibition and P73 Activation. Cancer Res. 76 (7), 1989–1999. 10.1158/0008-5472.CAN-14-2430 26759239

[B98] Qu X. Yu J. Bhagat G. Furuya N. Hibshoosh H. Troxel A. (2003). Promotion of Tumorigenesis by Heterozygous Disruption of the Beclin 1 Autophagy Gene. J. Clin. Invest. 112 (12), 1809–1820. 10.1172/JCI20039 14638851 PMC297002

[B99] Raufi A. G. (2021). Abstract 1006: Combination Therapy with MEK Inhibitors and a Novel Anti-neoplastic Drug, Imipridone ONC212, Demonstrates Synergy in Pancreatic Ductal Adenocarcinoma Cell Lines. Cancer Res. 81 (13 Suppl. ment), 1006.

[B100] Rebecca V. W. Nicastri M. C. McLaughlin N. Fennelly C. McAfee Q. Ronghe A. (2017). A Unified Approach to Targeting the Lysosome's Degradative and Growth Signaling Roles. Cancer Discov. 7 (11), 1266–1283. 10.1158/2159-8290.CD-17-0741 28899863 PMC5833978

[B101] Reggiori F. Ungermann C. (2017). Autophagosome Maturation and Fusion. J. Mol. Biol. 429 (4), 486–496. 10.1016/j.jmb.2017.01.002 28077293

[B102] Richardson C. Zhang S. Hernandez Borrero L. J. El-Deiry W. S. (2017). Small-molecule CB002 Restores P53 Pathway Signaling and Represses Colorectal Cancer Cell Growth. Cell Cycle 16 (18), 1719–1725. 10.1080/15384101.2017.1356514 28749206 PMC5602428

[B103] Roczniak-Ferguson A. Petit C. S. Froehlich F. Qian S. Ky J. Angarola B. (2012). The Transcription Factor TFEB Links mTORC1 Signaling to Transcriptional Control of Lysosome Homeostasis. Sci. Signal. 5, ra42. 10.1126/scisignal.2002790 22692423 PMC3437338

[B104] Ron D. (2002). Translational Control in the Endoplasmic Reticulum Stress Response. J. Clin. Invest. 110 (10), 1383–1388. 10.1172/JCI16784 12438433 PMC151821

[B105] Ronan B. Flamand O. Vescovi L. Dureuil C. Durand L. Fassy F. (2014). A Highly Potent and Selective Vps34 Inhibitor Alters Vesicle Trafficking and Autophagy. Nat. Chem. Biol. 10 (12), 1013–1019. 10.1038/nchembio.1681 25326666

[B106] Rouschop K. M. van den Beucken T. Dubois L. Niessen H. Bussink J. Savelkouls K. (2010). The Unfolded Protein Response Protects Human Tumor Cells during Hypoxia through Regulation of the Autophagy Genes MAP1LC3B and ATG5. J. Clin. Invest. 120 (1), 127–141. 10.1172/JCI40027 20038797 PMC2798689

[B107] Rutkowski D. T. Arnold S. M. Miller C. N. Wu J. Li J. Gunnison K. M. (2006). Adaptation to ER Stress Is Mediated by Differential Stabilities of Pro-survival and Pro-apoptotic mRNAs and Proteins. Plos Biol. 4, e374. 10.1371/journal.pbio.0040374 17090218 PMC1634883

[B108] Rzymski T. Milani M. Pike L. Buffa F. Mellor H. R. Winchester L. (2010). Regulation of Autophagy by ATF4 in Response to Severe Hypoxia. Oncogene 29 (31), 4424–4435. 10.1038/onc.2010.191 20514020

[B109] Samaras P. Tusup M. Nguyen-Kim T. D. L. Seifert B. Bachmann H. von Moos R. (2017). Phase I Study of a Chloroquine-Gemcitabine Combination in Patients with Metastatic or Unresectable Pancreatic Cancer. Cancer Chemother. Pharmacol. 80 (5), 1005–1012. 10.1007/s00280-017-3446-y 28980060

[B110] Shang L. Chen S. Du F. Li S. Zhao L. Wang X. (2011). Nutrient Starvation Elicits an Acute Autophagic Response Mediated by Ulk1 Dephosphorylation and its Subsequent Dissociation from AMPK. Proc. Natl. Acad. Sci. U S A. 108 (12), 4788–4793. 10.1073/pnas.1100844108 21383122 PMC3064373

[B111] Shao S. Li S. Qin Y. Wang X. Yang Y. Bai H. (2014). Spautin-1, a Novel Autophagy Inhibitor, Enhances Imatinib-Induced Apoptosis in Chronic Myeloid Leukemia. Int. J. Oncol. 44 (5), 1661–1668. 10.3892/ijo.2014.2313 24585095 PMC6904104

[B112] Showkat M. Beigh M. A. Bhat B. B. Batool A. Andrabi K. I. (2014). Phosphorylation Dynamics of Eukaryotic Initiation Factor 4E Binding Protein 1 (4E-BP1) Is Discordant with its Potential to Interact with Eukaryotic Initiation Factor 4E (eIF4E). Cel. Signal. 26 (10), 2117–2121. 10.1016/j.cellsig.2014.06.008 24975846

[B113] Siegel R. L. Miller K. D. Fuchs H. E. Jemal A. (2021). Cancer Statistics, 2021. CA A. Cancer J. Clin. 71 (1), 7–33. 10.3322/caac.21654 33433946

[B114] Sotelo J. Briceño E. López-González M. A. (2006). Adding Chloroquine to Conventional Treatment for Glioblastoma Multiforme: a Randomized, Double-Blind, Placebo-Controlled Trial. Ann. Intern. Med. 144 (5), 337–343. 10.7326/0003-4819-144-5-200603070-00008 16520474

[B115] Stein M. N. Bertino J. R. Kaufman H. L. Mayer T. Moss R. Silk A. (2017). First-in-Human Clinical Trial of Oral ONC201 in Patients with Refractory Solid Tumors. Clin. Cancer Res. 23 (15), 4163–4169. 10.1158/1078-0432.CCR-16-2658 28331050 PMC7595575

[B116] Strohecker A. M. Guo J. Y. Karsli-Uzunbas G. Price S. M. Chen G. J. Mathew R. (2013). Autophagy Sustains Mitochondrial Glutamine Metabolism and Growth of BrafV600E-Driven Lung Tumors. Cancer Discov. 3 (11), 1272–1285. 10.1158/2159-8290.CD-13-0397 23965987 PMC3823822

[B117] Suraweera A. Münch C. Hanssum A. Bertolotti A. (2012). Failure of Amino Acid Homeostasis Causes Cell Death Following Proteasome Inhibition. Mol. Cel. 48 (2), 242–253. 10.1016/j.molcel.2012.08.003 PMC3482661 22959274

[B118] Szegezdi E. Logue S. E. Gorman A. M. Samali A. (2006). Mediators of Endoplasmic Reticulum Stress-Induced Apoptosis. EMBO Rep. 7 (9), 880–885. 10.1038/sj.embor.7400779 16953201 PMC1559676

[B119] Takamura A. Komatsu M. Hara T. Sakamoto A. Kishi C. Waguri S. (2011). Autophagy-deficient Mice Develop Multiple Liver Tumors. Genes Dev. 25 (8), 795–800. 10.1101/gad.2016211 21498569 PMC3078705

[B120] Tallóczy Z. Jiang W. Virgin H. W. Leib D. A. Scheuner D. Kaufman R. J. (2002). Regulation of Starvation- and Virus-Induced Autophagy by the eIF2alpha Kinase Signaling Pathway. Proc. Natl. Acad. Sci. U S A. 99 (1), 190–195. 10.1073/pnas.012485299 11756670 PMC117537

[B121] Tang F. Hu P. Yang Z. Xue C. Gong J. Sun S. (2017). SBI0206965, a Novel Inhibitor of Ulk1, Suppresses Non-small Cell Lung Cancer Cell Growth by Modulating Both Autophagy and Apoptosis Pathways. Oncol. Rep. 37 (6), 3449–3458. 10.3892/or.2017.5635 28498429

[B122] Tian T. Li X. Zhang J. (2019). mTOR Signaling in Cancer and mTOR Inhibitors in Solid Tumor Targeting Therapy. Int. J. Mol. Sci. 20, 20. 10.3390/ijms20030755 PMC6387042 30754640

[B123] Tian X. Ahsan N. Lulla A. Lev A. Abbosh P. Dicker D. T. (2021). P53-independent Partial Restoration of the P53 Pathway in Tumors with Mutated P53 through ATF4 Transcriptional Modulation by ERK1/2 and CDK9. Neoplasia 23 (3), 304–325. 10.1016/j.neo.2021.01.004 33582407 PMC7890376

[B124] Vander Heiden M. G. Cantley L. C. Thompson C. B. (2009). Understanding the Warburg Effect: the Metabolic Requirements of Cell Proliferation. Science 324 (5930), 1029–1033. 10.1126/science.1160809 19460998 PMC2849637

[B125] Viale A. Pettazzoni P. Lyssiotis C. A. Ying H. Sánchez N. Marchesini M. (2014). Oncogene Ablation-Resistant Pancreatic Cancer Cells Depend on Mitochondrial Function. Nature 514 (7524), 628–632. 10.1038/nature13611 25119024 PMC4376130

[B126] Wagner J. Kline C. L. Ralff M. D. Lev A. Lulla A. Zhou L. (2017). Preclinical Evaluation of the Imipridone Family, Analogs of Clinical Stage Anti-cancer Small Molecule ONC201, Reveals Potent Anti-cancer Effects of ONC212. Cell Cycle 16 (19), 1790–1799. 10.1080/15384101.2017.1325046 28489985 PMC5628644

[B127] Wagner J. Kline C. L. Zhou L. Campbell K. S. MacFarlane A. W. Olszanski A. J. (2018). Dose Intensification of TRAIL-Inducing ONC201 Inhibits Metastasis and Promotes Intratumoral NK Cell Recruitment. J. Clin. Invest. 128 (6), 2325–2338. 10.1172/JCI96711 29533922 PMC5983321

[B128] Wang W. Kim S. H. El-Deiry W. S. (2006). Small-molecule Modulators of P53 Family Signaling and Antitumor Effects in P53-Deficient Human colon Tumor Xenografts. Proc. Natl. Acad. Sci. U S A. 103 (29), 11003–11008. 10.1073/pnas.0604507103 16835297 PMC1544164

[B129] White E. (2016). Autophagy and P53. Cold Spring Harb. Perspect. Med. 6, a026120. 10.1101/cshperspect.a026120 27037419 PMC4817743

[B130] White E. (2015). The Role for Autophagy in Cancer. J. Clin. Invest. 125 (1), 42–46. 10.1172/jci73941 25654549 PMC4382247

[B131] Whitney M. L. Jefferson L. S. Kimball S. R. (2009). ATF4 Is Necessary and Sufficient for ER Stress-Induced Upregulation of REDD1 Expression. Biochem. Biophys. Res. Commun. 379 (2), 451–455. 10.1016/j.bbrc.2008.12.079 19114033 PMC2656673

[B132] Wilson W. R. Hay M. P. (2011). Targeting Hypoxia in Cancer Therapy. Nat. Rev. Cancer 11 (6), 393–410. 10.1038/nrc3064 21606941

[B133] Wolpin B. M. Hezel A. F. Abrams T. Blaszkowsky L. S. Meyerhardt J. A. Chan J. A. (2009). Oral mTOR Inhibitor Everolimus in Patients with Gemcitabine-Refractory Metastatic Pancreatic Cancer. J. Clin. Oncol. 27 (2), 193–198. 10.1200/JCO.2008.18.9514 19047305 PMC2645085

[B134] Wolpin B. M. Rubinson D. A. Wang X. Chan J. A. Cleary J. M. Enzinger P. C. (2014). Phase II and Pharmacodynamic Study of Autophagy Inhibition Using Hydroxychloroquine in Patients with Metastatic Pancreatic Adenocarcinoma. Oncologist 19 (6), 637–638. 10.1634/theoncologist.2014-0086 24821822 PMC4041680

[B135] Wong P. M. Feng Y. Wang J. Shi R. Jiang X. (2015). Regulation of Autophagy by Coordinated Action of mTORC1 and Protein Phosphatase 2A. Nat. Commun. 6, 8048. 10.1038/ncomms9048 26310906 PMC4552084

[B136] Wood S. D. Grant W. Adrados I. Choi J. Y. Alburger J. M. Duckett D. R. (2017). In Silico HTS and Structure Based Optimization of Indazole-Derived ULK1 Inhibitors. ACS Med. Chem. Lett. 8 (12), 1258–1263. 10.1021/acsmedchemlett.7b00344 29259744 PMC5733266

[B137] Wu G. S. Burns T. F. McDonald E. R. Jiang W. Meng R. Krantz I. D. (1997). KILLER/DR5 Is a DNA Damage-Inducible P53-Regulated Death Receptor Gene. Nat. Genet. 17 (2), 141–143. 10.1038/ng1097-141 9326928

[B138] Yamamoto K. Venida A. Yano J. Biancur D. E. Kakiuchi M. Gupta S. (2020). Autophagy Promotes Immune Evasion of Pancreatic Cancer by Degrading MHC-I. Nature 581 (7806), 100–105. 10.1038/s41586-020-2229-5 32376951 PMC7296553

[B139] Yang A. Herter-Sprie G. Zhang H. Lin E. Y. Biancur D. Wang X. (2018). Autophagy Sustains Pancreatic Cancer Growth through Both Cell-Autonomous and Nonautonomous Mechanisms. Cancer Discov. 8 (3), 276–287. 10.1158/2159-8290.CD-17-0952 29317452 PMC5835190

[B140] Yang A. Kimmelman A. C. (2014). Inhibition of Autophagy Attenuates Pancreatic Cancer Growth Independent of TP53/TRP53 Status. Autophagy 10 (9), 1683–1684. 10.4161/auto.29961 25046107 PMC4206544

[B141] Yang A. Rajeshkumar N. V. Wang X. Yabuuchi S. Alexander B. M. Chu G. C. (2014). Autophagy Is Critical for Pancreatic Tumor Growth and Progression in Tumors with P53 Alterations. Cancer Discov. 4 (8), 905–913. 10.1158/2159-8290.CD-14-0362 24875860 PMC4125497

[B142] Yang S. Wang X. Contino G. Liesa M. Sahin E. Ying H. (2011). Pancreatic Cancers Require Autophagy for Tumor Growth. Genes Dev. 25 (7), 717–729. 10.1101/gad.2016111 21406549 PMC3070934

[B143] Ye J. Kumanova M. Hart L. S. Sloane K. Zhang H. De Panis D. N. (2010). The GCN2-ATF4 Pathway Is Critical for Tumour Cell Survival and Proliferation in Response to Nutrient Deprivation. EMBO J. 29 (12), 2082–2096. 10.1038/emboj.2010.81 20473272 PMC2892366

[B144] Young C. D. Arteaga C. L. Cook R. S. (2015). Dual Inhibition of Type I and Type III PI3 Kinases Increases Tumor Cell Apoptosis in HER2+ Breast Cancers. Breast Cancer Res. 17, 148. 10.1186/s13058-015-0656-2 26637440 PMC4670529

[B145] Young T. M. Reyes C. Pasnikowski E. Castanaro C. Wong C. Decker C. E. (2020). Autophagy Protects Tumors from T Cell-Mediated Cytotoxicity via Inhibition of TNFα-Induced Apoptosis. Sci. Immunol. 5, 5. 10.1126/sciimmunol.abb9561 33443027

[B146] Yue Z. Jin S. Yang C. Levine A. J. Heintz N. (2003). Beclin 1, an Autophagy Gene Essential for Early Embryonic Development, Is a Haploinsufficient Tumor Suppressor. Proc. Natl. Acad. Sci. U S A. 100 (25), 15077–15082. 10.1073/pnas.2436255100 14657337 PMC299911

[B147] Yun Z. Lin Q. (2014). Hypoxia and Regulation of Cancer Cell Stemness. Adv. Exp. Med. Biol. 772, 41–53. 10.1007/978-1-4614-5915-6_2 24272353 PMC4043215

[B148] Zhang S. Zhou L. Hong B. van den Heuvel A. P. Prabhu V. V. Warfel N. A. (2015). Small-Molecule NSC59984 Restores P53 Pathway Signaling and Antitumor Effects against Colorectal Cancer via P73 Activation and Degradation of Mutant P53. Cancer Res. 75 (18), 3842–3852. 10.1158/0008-5472.CAN-13-1079 26294215 PMC4573895

[B149] Zhang S. (2018). Abstract 1866: Small Molecule NSC59984 Is a Radio-Sensitizer Dependent on ERK2 and DDR but Independent of Wild-type P53. Cancer Res. 78 (13 Suppl. ment), 1866.

[B150] Zhang S. (2017). Abstract 1892: Small Molecule NSC59984 Prevents Cancer Cell Migration and Invasion. Cancer Res. 77 (13 Suppl. ment), 1892. 28108509

[B151] Zhang S. (2017). Abstract 2156: NSC59984 Induces Mutant P53 Degradation via Activating ERK2 Pathway-MDM2 axis. Cancer Res. 77 (13 Suppl. ment), 2156.

[B152] Zhang Y. Morgan M. J. Chen K. Choksi S. Liu Z. G. (2012). Induction of Autophagy Is Essential for Monocyte-Macrophage Differentiation. Blood 119 (12), 2895–2905. 10.1182/blood-2011-08-372383 22223827 PMC3327464

[B153] Zou Z. Yuan Z. Zhang Q. Long Z. Chen J. Tang Z. (2012). Aurora Kinase A Inhibition-Induced Autophagy Triggers Drug Resistance in Breast Cancer Cells. Autophagy 8 (12), 1798–1810. 10.4161/auto.22110 23026799 PMC3541289

